# Dynamic Bonds Reinforced Polyamide Elastomer for Biomedical Orthosis

**DOI:** 10.1002/advs.202504395

**Published:** 2025-05-20

**Authors:** Zhen Li, Peiyao Yan, Hao Wang, Yuancheng Zhang, Junhua Kong, Wei Zhao, Xin Li, Xiaomeng Zhang, Zhe Cui, Peng Fu, Xinchang Pang, Minying Liu, Chaobin He

**Affiliations:** ^1^ School of Materials Science and Engineering Zhengzhou University Zhengzhou 450001 China; ^2^ Department of Materials Science and Engineering National University of Singapore Singapore 117575 Singapore; ^3^ Zhengzhou University Industrial Technology Research Institute Co., Ltd. Zhengzhou 450001 China; ^4^ Henan Key Laboratory of Advanced Nylon Materials and Application Zhengzhou University Zhengzhou 450052 China; ^5^ Henan Tuoren Medical Device Co. Ltd. Xinxiang 453400 China; ^6^ Institute of Materials Research and Engineering Agency for Science Technology and Research (A∗STAR) Singapore 138634 Singapore

**Keywords:** 4D printing, interfacial reinforcing, multiple dynamic bonds, poly(urethane‐urea‐amide) elastomer, shape memory polymer

## Abstract

4D printing of shape memory polymers (SMPs) allows the 3D‐printed structures to have adjustable shapes, properties, and functionalities, paving the way for intelligent devices and multifunctional applications. However, 4D‐printed SMPs face challenges due to mechanical anisotropy and mediocre shape memory performance hampered by weak interlayer adhesion. This study innovatively integrates shape memory polyamide elastomer with 4D printing technology to develop a multifunctional intelligent orthosis. Here, a dynamic bonds (DBs) reinforced shape memory polyamide elastomer is developed using a twin‐screw extruder through reactive extrusion. Dynamic covalent networks are introduced into polyamide elastomer, which enhances interlayer adhesion in 4D‐printed objects by utilizing combined effects of multiple dynamic covalent bonds (DCBs) and hierarchical hydrogen bonds (DHBs), leading to the reduction of mechanical anisotropy and improvement of the mechanical and shape memory properties of the 4D printouts. 4D‐printed objects demonstrated excellent macroscopic shape memory and reconfiguration, showcasing the versatility of this material, and the application for spinal orthosis is also demonstrated.

## Graphical abstract

A dynamic bonds (DBs) reinforced shape memory polyamide elastomer is developed using reactive extrusion. Dynamic covalent networks enhance interlayer adhesion in 4D‐printed objects by utilizing the combined effects of multiple DBs, reducing mechanical anisotropy and improving the mechanical and shape memory properties. 4D‐printed objects demonstrate excellent macroscopic shape memory and reconfiguration, and the application for spinal orthosis is also demonstrated.

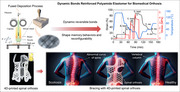

## 1 Introduction

4D printing, as an intelligent additive manufacturing technology, introduces the dimension of time to 3D printing,^[^ ^1^ ^]^ meaning the shape, properties or functionality of 3D‐printed structures can self‐transform when exposed to predetermined stimuli, such as moisture, heat,^[^ ^2^ ^]^ light,^[^ ^3^ ^]^ electricity,^[^ ^4^ ^]^ and magnetism,^[^ ^5^ ^]^ etc. In the context of carbon neutrality and artificial intelligence, 4D printing offers key benefits over traditional methods like injection molding. These include smarter, personalized designs, less material waste, no need for expensive molds, and faster production from design to final product.^[^ ^6^ ^]^ The 4D‐printed orthosis, an individually designed or customized device, is applied to the external part of the body to provide support and protection for that particular area of the musculoskeletal system.^[^ ^7^ ^]^ Spinal orthoses produced using additive manufacturing show great potential for obtaining patient‐specific solutions in clinical applications, reducing manual operations, time consumption, and material waste.^[^ ^8^ ^]^ The predominant use of metal and leather in orthotic devices is gradually being replaced by lightweight thermoplastics and foams, which facilitate well‐designed, breathability, and comfortable options for patients.^[^ ^9^ ^]^

Among 4D‐printed polymers, shape memory polymers (SMPs) are the most widely used stimuli‐responsive materials, ideal for the intelligent manufacturing of biomedical orthoses.^[^ ^10^ ^]^ SMPs can hold a temporary shape after an external force is applied and then return to their original shape when triggered by external stimuli.^[^ ^11^ ^]^ Due to their large deformation capability, high sensitivity, low cost, and low density,^[^ ^12^ ^]^ 4D‐printed SMPs have gained significant attention in both academia and industry since their introduction in 2013.^[^ ^13^ ^]^ As smart materials, SMPs show great potential for developing next‐generation 4D‐printed preforms for applications like tissue engineering, health monitoring, and soft robotics.^[^ ^12^ ^]^ SMPs can be categorized as amorphous, semicrystalline, and liquid crystalline elastomer based on their crystalline properties.^[^ ^14^ ^]^ However, most 4D‐printed SMPs (e.g., thermoplastic polyurethane,^[^ ^15^ ^]^ polycaprolactone,^[^ ^16^ ^]^ polylactic acid,^[^ ^17^ ^]^ polycarbonate,^[^ ^4^ ^]^ polyamide,^[^ ^18^ ^]^ thermoplastic polyamide elastomer,^[^ ^19^ ^]^ et al.) face challenges such as low mechanical strength due to the rapid cooling of the printed strands.^[^ ^20^ ^]^ Additionally, 4D printing without pressure can create pores or defects in the structure, and traditional thermoplastic SMPs have weak bonding between layers, especially in the vertical direction, because the layers mainly rely on weak van der Waals forces between the polymer chains.^[^ ^21^ ^]^ Bodaghi et al.^[^ ^22^ ^]^ conducted experimental and numerical studies on the thermos‐mechanical behaviors of 4D‐printed self‐morphing structures under large‐strain and compressive loading, revealing buckling instability characteristics, which were subsequently leveraged to optimize the printing process and enhance the mechanical performance of 4D‐printed SMPs. Levenhagen^[^ ^23^ ^]^ introduced a surface‐segregating additive into polylactide, which increased the inter‐layer maximum stress by 40% after FDM printing. However, the additive was not effective enough to reduce anisotropy between layers.

To address the challenges mentioned above, several extrinsic and intrinsic approaches have recently been developed, focusing on interfacial adhesion mechanisms (refer to Table S1, Supporting Information, and related introduction). Extrinsic method scan improves layer bonding but significantly increases manufacturing costs and reduces dimensional accuracy.^[^ ^24^ ^]^ Attention has shifted toward designing polymer chemical structures and optimizing 4D printing parameters. One promising approach is the introduction of dynamic bonds into the polymer matrix.^[^ ^25^ ^]^ Dynamic covalent bonds (DCBs), first proposed by Lehn and co‐workers^[^ ^26^ ^]^ in 1999, create reversible polymer networks known as “covalent adaptable network (CAN)”,^[^ ^27^ ^]^ “vitrimer”^[^ ^28^ ^]^ or “thermadapt polymer”.^[^ ^29^ ^]^ These bonds can selectively and reversibly break and reform in response to stimuli like temperature, catalysts, or light, allowing dynamic equilibrium under non‐equilibrium conditions, which improves interlayer bonding and reduces mechanical anisotropy. Lu and co‐workers^[^ ^24^ ^]^ introduced dynamic urea bonds into polyurethane, enhancing interlayer adhesion in FDM printing through heat activation and topological rearrangement. Repeated heating of the printed parts also reduces internal defects and improves mechanical properties. Walter^[^ ^30^ ^]^ introduced dynamic Diels‐Alder bonds into thermoset polymers, allowing re‐crosslinking and processing at lower temperatures. However, the improvement in mechanical properties of the printed objects was still limited. While previous studies have established the use of shape‐memory polymers (SMPs) combined with dynamic covalent and hydrogen bonds for 4D printing, limited attention has been paid to polyamide elastomers reinforced with multiple dynamic bonds, particularly for spinal orthosis applications. In addition, we propose a novel integrated strategy combining reactive extrusion and 4D printing to fabricate dynamic bond‐reinforced polyamide elastomers. This method provides a more cost‐effective and efficient pathway for additive manufacturing, especially suitable for spinal orthotic devices that require complex geometries, patient‐specific customization, and an optimal balance between mechanical rigidity for corrective support and elasticity and breathability for comfortable wearability. This work innovatively proposes the application of the developed polyamide elastomer in smart wearable and biomedical orthotic devices, broadening the application scope of 4D‐printable polyamide materials and providing new insights into intelligent manufacturing and spinal orthosis design.

In this work, a dynamic bonds (DBs) reinforced shape memory poly(urethane‐urea‐amide) elastomer (PUUA‐DB) was developed using a twin‐screw extruder through reactive extrusion. Dynamic covalent networks were introduced into polyamide elastomer, which enhances interlayer adhesion in 4D‐printed objects by utilizing synergistic effects of multiple dynamic covalent bonds (DCBs) and hierarchical hydrogen bonds (DHBs), leading to the reduction of mechanical anisotropy and improvement of the mechanical and shape memory properties of the 4D printouts. Comparison with conventional materials suffers from poor interlayer fusion due to the absence of self‐healing mechanisms. This material, characterized by enhanced interlayer bonding and mechanical strength, is essential for providing sufficient structural support and shape fixation in spinal orthoses. Improved shape memory performance ensures more accurate and reliable shape retention and recovery, which are critical for the precise fit and corrective function of spinal orthoses. Reduced mechanical anisotropy is highly beneficial for spinal braces subjected to multi‐directional forces. The ability to reconfigure permanent shapes extends the service life and reusability of spinal orthotic devices. The dynamic mechanisms of DHBs and DCBs were studied using in situ FTIR, thermomechanical analysis, and morphological analysis, revealing insights into how these interactions enhance the mechanical properties and enable multiple/reversible two‐way shape memory in the 4D‐printed material. 4D‐printed objects demonstrated excellent one‐way dual shape memory, quadruple shape memory, reversible two‐way shape memory, and reconfiguration, showcasing the versatility of this material. 4D‐printed spinal orthosis pattern using our synthesized polymer can fit human body structure conformally by changing from temporary 1D shape to its original 2D shape via shape memory effect only triggered by the body temperature. The abnormal spine curvature of scoliosis was effectively alleviated by the treatment with 4D‐printed spinal orthosis after several iterations. The novel PUUA‐DB material not only expands the range of 4D‐printable SMPs but also shows potential applications in soft robotics and the biomedical field.

## 2 Results

### 2.1 Synthesis and Characterization of PUUA‐DBs

**Figure 1:**
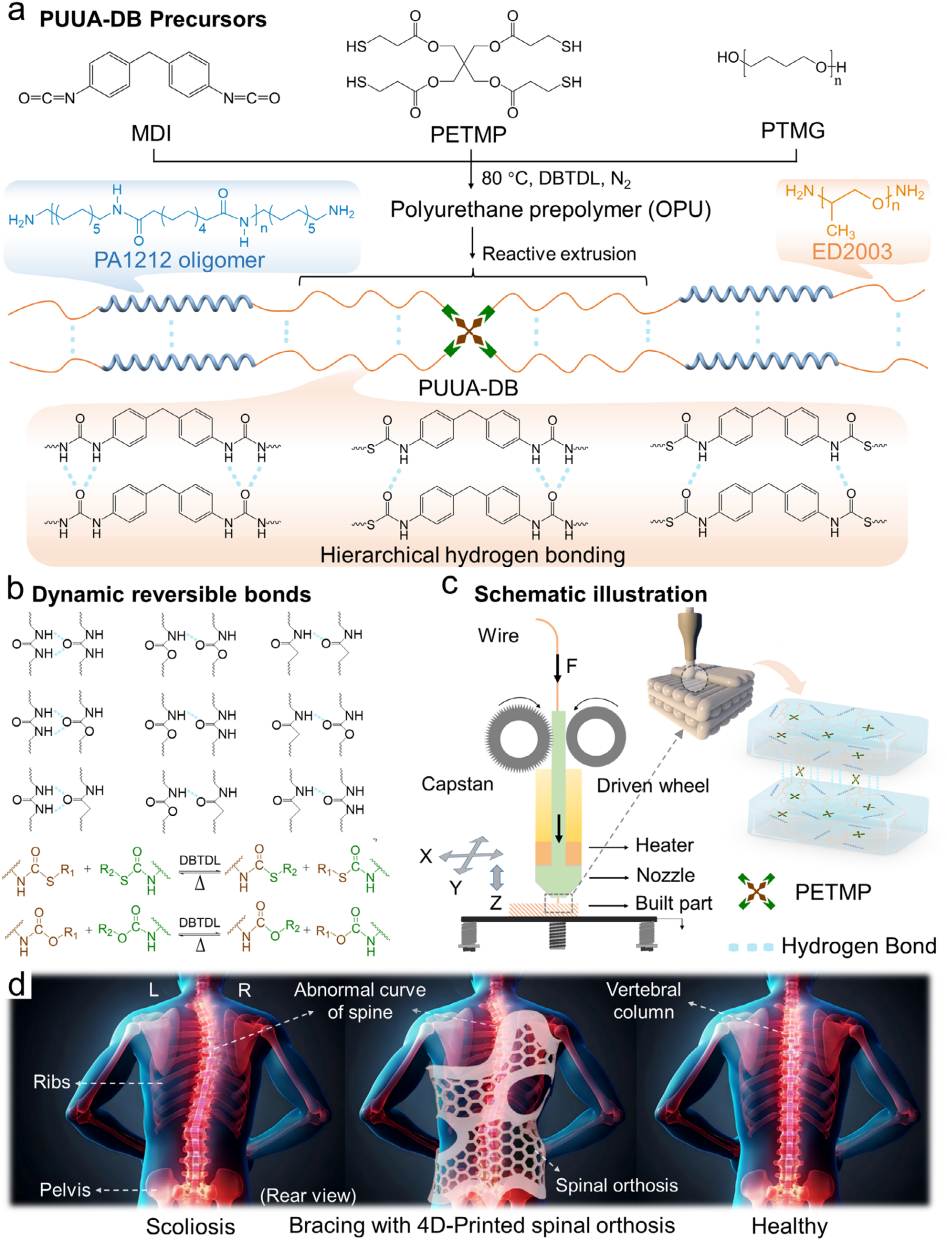
Chemical design and functional application of 4D‐printed shape memory poly(urethane‐urea‐amide) elastomers (PUUA‐DBs). a) Synthetic route and b) interfacial reinforcing mechanism of PUUA‐DB containing hierarchical hydrogen bonding and multiple dynamic covalent bonds. c) 4D printing process of PUUA‐DB extruded through heated nozzle above the reversed reaction temperature of dynamic covalent bonds and deposited on a substrate to form a 4D printout reinforced by interlayer multiple dynamic bonds. d) Schematic of 4D‐printed spinal orthosis for bracing scoliosis patients.

Poly(urethane‐urea‐amide) elastomers (PUUA‐DBs) were synthesized using the isocyanate method through reactive extrusion. First, an isocyanate‐terminated polyurethane prepolymer (OPU) containing DCBs was synthesized using polytetramethylene ether glycol (PTMG), 4,4′‐methylenebis(phenyl isocyanate) (MDI), and pentaerythritol terakis(3‐mercaptopropionate) (PETMP). PUUA‐DB was then prepared through block copolymerization of the OPU, polyether amine (ED2003), and amino‐terminated polyamide 1212 (PA1212) in a twin‐screw extruder using reactive extrusion (**Figure** **1**a). The PUUA‐DBs were further extruded into wires with a diameter of 1.75 ± 0.05 mm for FDM printing. In this process, the ─OH groups of PTMG and the ─SH groups of PETMP underwent a nucleophilic addition reaction with the ─NCO groups of MDI, forming carbamate and thiocarbamate groups, respectively. Additionally, the ─NH_2_ groups of ED2003 and PA1212 reacted with the ─NCO groups of MDI to form urea groups (Figure S2, Supporting Information, and discussion). PUUA‐DBs possess inherent advantages due to abundant and hierarchical hydrogen‐bonding interactions (Figure 1b), including single hydrogen bonds from carbamate and amide groups, as well as stronger dual bifurcated hydrogen bonds provided by urea groups.^[^ ^31^ ^]^ The dynamic covalent crosslinking networks (CANs) in the polymer, formed through reversible thiocarbamate, carbamate, and hydrogen bonds, undergo topological rearrangement when exposed to changes in temperature or catalyst (DBTDL). This process helps the system reach dynamic equilibrium, giving PUUA‐DB unique viscoelastic and plastic properties, along with controllable viscosity and fluidity, making it well‐suited for extrusion‐based additive manufacturing. It is anticipated that 3D printing above the dissociation temperature of multiple dynamic bonds will enhance polymer chain diffusion and entanglement between 3D‐printed layers (Figure 1c), thereby improving the mechanical properties and reducing mechanical anisotropy of 4D‐printed spinal orthosis (Figure 1d). To explore the optimal formulation and investigate the structure‐performance relationship for 4D printing, four sets of PUUA‐DBs were synthesized with varying ED2003 contents (0–11 mol%) and different crosslinkers (Table S2, Supporting Information).

### 2.2 Multiple Dynamic Bonds of PUUA‐DBs

**Figure 2:**
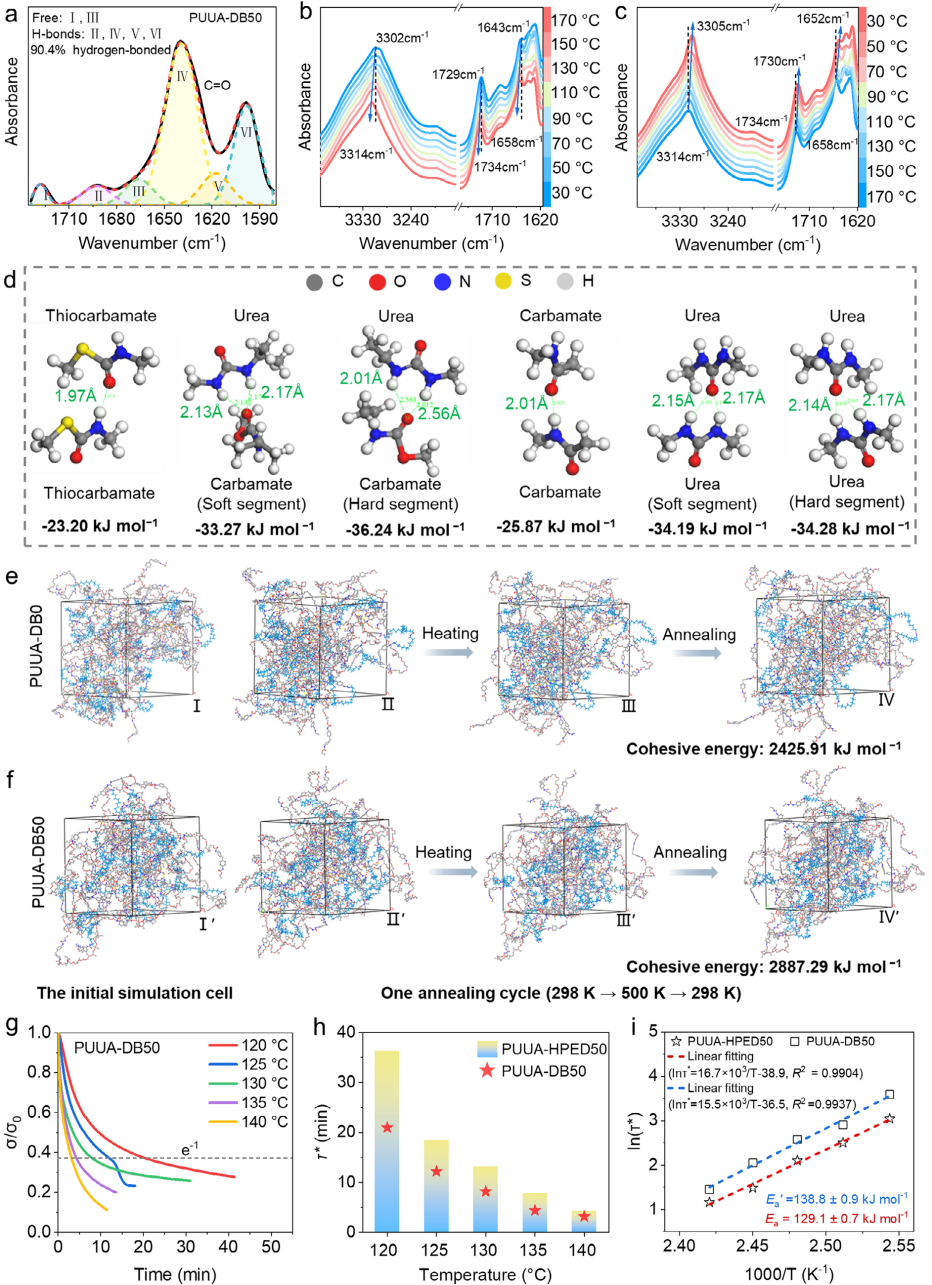
Thermal reversibility of multiple dynamic bonds in PUUA‐DBs and its chemical simulation analysis. a) The deconvoluted subpeaks of FTIR spectra in the C═O stretching vibration region for PUUA‐DB50. b,c) Variable‐temperature FTIR spectra of PUUA‐DB50. d) Interaction energy (∆*E*_H_) and bond length (*L*) for different hydrogen bonds calculated by quantum chemical calculations. The ball‐and‐stick model represents atoms and chemical bonds, while dotted lines highlighted the hydrogen bonds. The equilibrium conformation of e) PUUA‐DB0 and f) PUUA‐DB50 elastomers in simulation cells by MD simulations (I, I′) and the conformation change in one annealing cycle including a linear heating process from 298 K to 500 K (II/II′ → III/III′) and a linear cooling process from 500 to 298 K (III/III′ → IV/IV′). Indigo‐blue line model indicates PA1212 segments, and gray, red, blue, yellow, and white line models represent carbon, oxygen, nitrogen, sulfur, and hydrogen atoms, respectively. Normalized stress versus time curves of g) PUUA‐DB50. h) Characteristic relaxation time (τ*) of PUUA‐HPED50 and PUUA‐DB50 at different temperatures. i) Characteristic relaxation time τ* versus reciprocal temperature *T* curves of PUUA‐HPED50 and PUUA‐DB50 and the fitted line by Arrhenius equation.

To investigate the influence of multiple dynamic bonds on PUUA‐DB elastomers and 4D printing performance, we analyzed the bonding energy (∆*E*_H_) and cohesive energy (*E*_cohesive_) of different types of hydrogen bonds in polymers using Gaussian fitting, quantum chemical calculations, and molecular dynamics simulations. First, the intermolecular hydrogen‐bond interaction was revealed by the deconvolution of C═O peaks in the FTIR spectra of the PUUA‐DBs (Figure 2a; Figure S3a–c, Supporting Information and related discussion). Quantitative analysis indicated that the fractions of hydrogen‐bonded C═O in the PUUA‐HPED50, PUUA‐DB0, PUUA‐DB25, and PUUA‐DB50 elastomers are 91.1%, 83.9%, 89.5%, and 90.4%, respectively (Table S5, Supporting Information). The increase in ED2003 content led to a rise in the number of C═O forming hydrogen bonds in PUUA‐DB, suggesting a gradual increase in hydrogen bonding. This can be attributed to the tendency of urea bonds formed with ED2003 to establish stronger dual bifurcated hydrogen‐bonding interactions (Figure 1a). The reversible evolution of dynamic hydrogen bonds in PUUA‐DB50 during temperature increase was analyzed (**Figure** **2**b,c), revealing how the hydrogen bonds respond and adjust to thermal changes. The N─H stretching vibration peak of the urea groups blue‐shifted from 3306 to 3315 cm^−1^ with an increase in temperature from 30 to 170 °C. Meanwhile, the C═O stretching vibrations of bifurcated hydrogen‐bonded urea and single hydrogen‐bonded carbamate shifted toward higher wavenumber at 1726 cm^−1^ and 1633 cm^−1^, respectively. This was attributed to the dissociation of hydrogen bonds, caused by higher energy required for the C═O stretching vibration of urea and carbamate groups, leading to an increase in the number of free hydrogen bonds.^[^ ^32^ ^]^ The thermal reversibility of hydrogen bonds was confirmed as the absorption peaks of the N─H and C═O shifted back to the lower wavenumbers upon cooling to 30 °C.^[^ ^33^ ^]^

The different hydrogen‐bonding interactions were simulated by quantum chemical calculations (Figure 2d). The calculation results showed that the interaction energy (*∆E*_H_) increased in the following order: thiocarbamate‐thiocarbamate, carbamate‐carbamate, urea‐carbamate, and urea‐urea interactions. The bond length (*L*) of hydrogen bonds was consistent with the typical range (1.95–2.16 Å), which is characteristic of stable hydrogen bonds.^[^ ^34^ ^]^ Additionally, the introduction of ED2003 into PUUA‐DB50 creates dual bifurcated hydrogen bonds with longer *L* and stronger interactions between urea groups.^[^ ^35^ ^]^ Furthermore, all‐atom molecular dynamics (MD) simulations were performed to analyze the intramolecular and intermolecular cohesive energy (*E*_cohesive_) of PUUA‐DB0 and PUUA‐DB50 after reaching equilibrium in the simulation cells (Figure 2e; Figure S4a,b, Supporting Information). The average *E*_cohesive_ per chain for PUUA‐DB0 and PUUA‐DB50 was calculated as 2425.91 and 2887.25 kJ mol^−1^, respectively (Figure S4c,d and Equation S2, Supporting Information). This indicated that the intramolecular and intermolecular interactions of PUUA‐DB50, including hydrogen bonds and non‐covalent bonds, are significantly stronger than those of PUUA‐DB0, owing to the highly organized and packed structures. This strength arises from the symmetrical nature of MDI, which is generated by bifurcated hydrogen‐bonding pairs.^[^ ^35^, ^36^ ^]^ Additionally, the simulated distribution of PA1212 segments (indigo–blue) exhibited significant microphase separation in PUUA‐DB50.^[^ ^32^, ^37^ ^]^

Furthermore, stress relaxation experiments were conducted to characterize the activation energy (*E*_a_) of the dynamic covalent crosslinking network (Figure S5, Supporting Information; Figure 2g). The PUUA‐HPED50 network contains dynamic carbamate bonds, while the PUUA‐DB50 network comprises dual dynamic covalent bonds (carbamate and thiocarbamate bonds). In a Maxwell viscoelastic fluid model, the relaxation time is defined as the time required for the instantaneous stress decreasing to 36.8% (e^−1^) of its initial stress.^[^ ^28^ ^]^ The results indicate that the stress relaxation rates of PUUA‐HPED50 and PUUA‐DB50 both raise up with the increase of temperature from 120 to 140 °C (Figure 2h), and the characteristic relaxation time (τ*) of PUUA‐DB50 consistently remains lower than that of PUUA‐HPED50 during temperature variations. Furthermore, activation energy of DCBs was calculated by fitting ln(τ*) versus 1000 T^−1^ (Arrhenius equation, Equation S3, Supporting Information). The activation energy of PUUA‐DB50 (*E*_a_) is 129.1 ± 0.7 kJ mol^−1^, which is lower than activation energy of PUUA‐HPED (*E*_a_′) of 138.8 ± 0.9 kJ mol^−1^ (Figure 2i). These results indicated that the movement of polymer chains and the exchange rate of dynamic thiocarbamate and carbamate bonds in the polymer networks are dependent on temperature and are promoted by the catalyst DBTDL. The interfacial enhancement mechanisms for improving the mechanical and shape memory properties, as well as reconstructing the permanent shape, are further studied by examining the synergy of multiple dynamic bonds in 4D printout.

### 2.3 Thermal and Mechanical Properties of PUUA‐DBs

**Figure 3:**
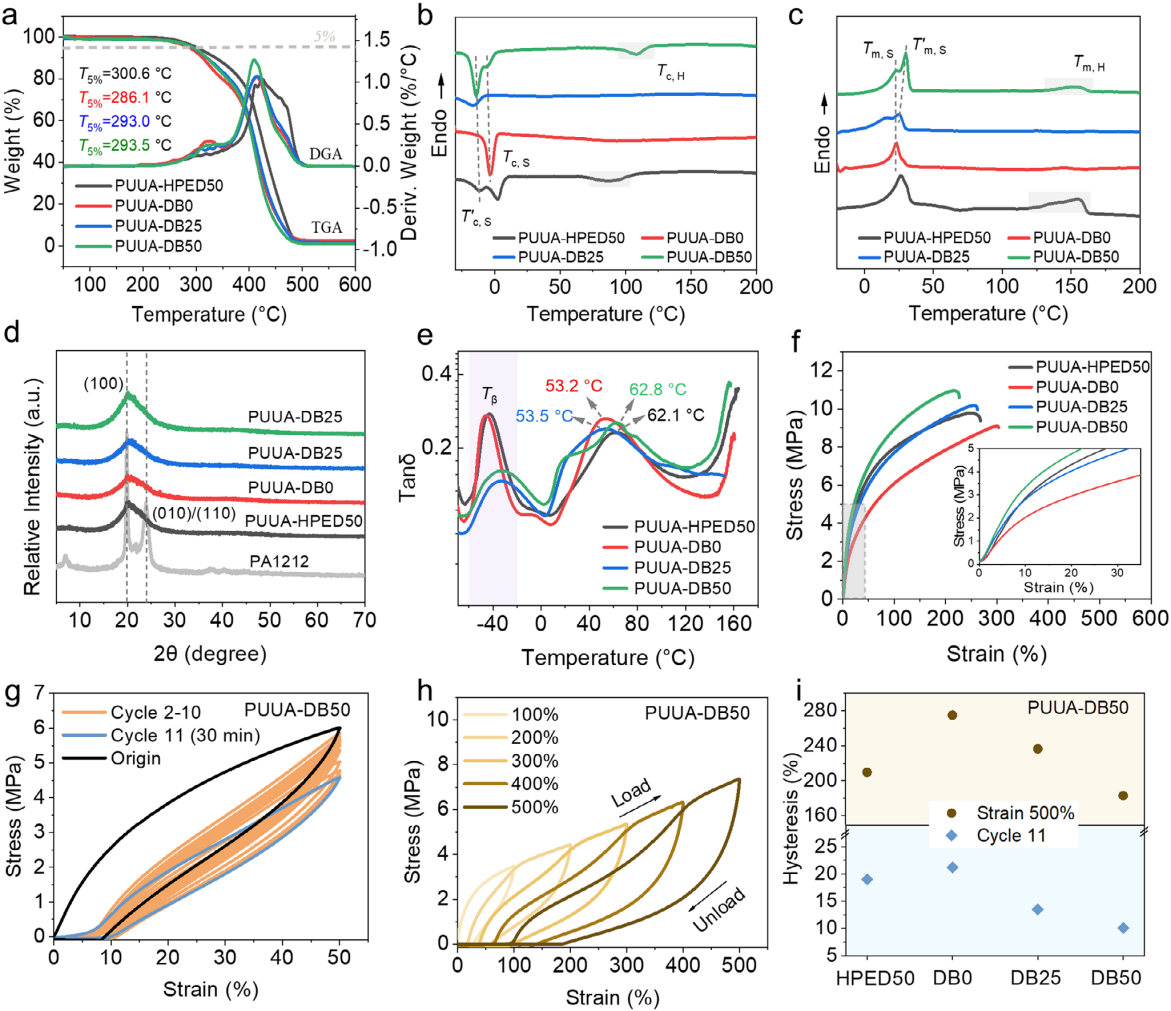
Thermal and mechanical performance of 4D printable PUUA‐DBs. a) TGA and DTG curves of PUUA‐DBs. b) DSC cooling and c) secondary heating curves of PUUA‐DB wires. d) XRD spectra of PUUA‐DBs. e) Loss factor (tan δ) of PUUA‐DBs. f) Stress–strain curves of PUUA‐DBs, insert stress–strain curves at 0–35% strain. g) Stress–strain curves of the loading‐unloading cycle for PUUA‐DB50. h) Stress–strain curves of the loading‐unloading cycle at different maximum tensile strains for PUUA‐DB50. i) Hysteresis ratio in the 11th test cycle and during the 500% maximum tensile strain cycle of PUUA‐DBs.

The thermal degradation, melt crystallization, and dynamic thermomechanical properties of PUUA‐DB wires were analyzed to investigate their thermal stability, FDM printing temperature window, and shape memory response temperature. As shown in **Figure** **3**a, all samples exhibited an initial degradation temperature (*T*_d, 5%_) higher than 265 °C and underwent obvious two‐step thermal degradation, involving the thermal degradation of polyurethane and PA1212 segments^[^ ^38^ ^]^ (Table S6, Supporting Information and related discussion). This indicated that PUUA‐DBs possess excellent thermal stability and a wide temperature range suitable for FDM printing. The crystallization and melting behaviors of PUUA‐DBs significantly influence the shape memory performance. The DSC results revealed that the PTMG segments in pristine PUUA‐DB0 sample exhibited crystallization temperature of −3.7 °C and melting transition temperature of 23.3 °C. These transitions disappeared in PA1212 segments due to hindrance of crystallization caused by crosslinker PETMP or HPED (Figure S6, Supporting Information; Figure 3b,c).^[^ ^39^ ^]^ As shown in Figure 3d, the XRD spectrum (collected at 25.0 ± 0.5 °C) of PA1212 oligomer showed two diffraction peaks at 20 and 24° in (100) and (010/110) crystal planes, respectively, indicating a typical 𝛼‐crystal of the triclinic structure. However, only a peak at 20.2° appeared in XRD spectrum of PUUA‐DBs, indicating an unstable pseudo‐hexagonal γ‐crystal structure. That was caused by the high flexibility of polyether segments in PTMG and ED2003, which made the hydrogen‐bonded polyamide segments prone to torsion and crystal transition.^[^ ^40^ ^]^

Additionally, the PTMG segments also restricted the crystallization of PA1212. Two distinct crystallization/melting peaks of the soft segments were observed in PUUA‐DB50 with the increase of ED2003 content, corresponding to the crystallization and melting temperature of PTMG (*T*_c,S_ = −6.0 °C, *T*_m,S_ = 23.0 °C) and ED2003 (*T′*_c,S_ = −14.0 °C, *T′*_m,S_ = 30.0 °C) segments, respectively (Table S7, Supporting Information). Intriguingly, the crystallization and melting peaks of PA1212 segment (*T*_c,H_ and *T*_m,H_) were observed at the 11 mol% ED2003 in both PUUA‐DB50 and PUUA‐HPED50. This phenomenon can be attributed to the stronger bifurcated hydrogen bonding interactions formed by urea bonds through reaction between ED2003 and MDI. These interactions enhanced the intermolecular hydrogen bonding interactions in PA1212 amorphous phase, promoting the formation of crystalline domains, as supported by the simulated segmental distribution from molecular dynamics simulation (Figure 2e,f). PUUA‐DBs exhibited similar rheological curves (Figure S7a, Supporting Information and discussion). As shown in Figure S7a (Supporting Information), with increasing temperature, the storage modulus (*E′*) decreased, while the *T*_g_ increased (Figure 3e). This phenomenon can be attributed to the increase in bifurcated hydrogen bond content, which formed stronger intermolecular hydrogen bond interactions and hindered the mobility of PA1212 segments in amorphous phase, and resulting in a rise of *T*_g_. Compared to the pristine PUUA‐DB0 sample, PUUA‐DB50 with 11.0 mol% ED2003 exhibited a significant increase in both *T*_β_ and *T*_g_ (Table S7, Supporting Information). This increase was attributed to the dual bifurcated hydrogen‐bonded urea groups, which possess relatively strong interactions and hinder the movement of amorphous soft segments and PA1212 molecular chains (Figure 2d).

As shown in Figure 3f and Figure S7 (Supporting Information), the mechanical properties and tensile cyclic stability of PUUA‐DBs were significantly improved with increasing ED2003 content. Among these, the tensile strength and elongation at break changed from 9.0 ± 0.2 MPa and 303.1 ± 7.0% for PUUA‐DB0 to 11.0 ± 0.3 MPa and 226.4 ± 6.0% for PUUA‐DB50, which can be attributed to the bifurcated hydrogen bonds formed by urea groups. Ten consecutive cyclic tensile tests with maximum tensile strain of 50% (Figure S8a–c, Supporting Information; Figure 3g) and different maximum tensile strains (100–500%) (Figure S8d–f, Supporting Information; Figure 3h) were performed. Due to the low strain rate (5 mm min^−1^), stretchability can reach up to 500% under cyclic loading. The eleventh cycle was performed after a stress relaxation for 30 min at room temperature. The results showed that all samples existed large hysteresis loop in the first cycle, indicating significant energy dissipation. However, the area of hysteresis loop decreased after the second cycle because only a small amount of hydrogen bonds reformed in the short time between adjacent cycles. With the increase in ED2003 content, both the hysteresis percentage of the 11th cycle and the cycle at a maximum tensile strain of 500% gradually decreased (Figure 3i). This was due to the increased hydrogen bonds density, which restricted the chain movement and resulted in enhanced elasticity. After a 30‐minute relaxation period, the stress‐strain curves of cycle 11 overlapped with the cycle 2 to 10, but the hysteresis percentage only recovered partially. This partial recovery was attributed to the chemically crosslinked network, which impeded the chain movement and, consequently, reconstruction of hydrogen bonds.

### 2.4 4D Printing of PUUA‐DB Wires

The viscosity and melt flow index of PUUA‐DBs have a significant impact on the efficiency of 3D printing. Intrinsic viscosity [η] and melt flow rate (*MFR*) of PUUA‐DB wires (Figure S9a, Supporting Information) were measured for evaluating their printability. PUUA‐DB50 exhibited good FDM printability thanks to its low viscosity ([η] = 1.1) and good melt fluidity (*MFR* = 20.1 g min^−1^) (Figure S10 and Table S8, Supporting Information).

**Figure 4:**
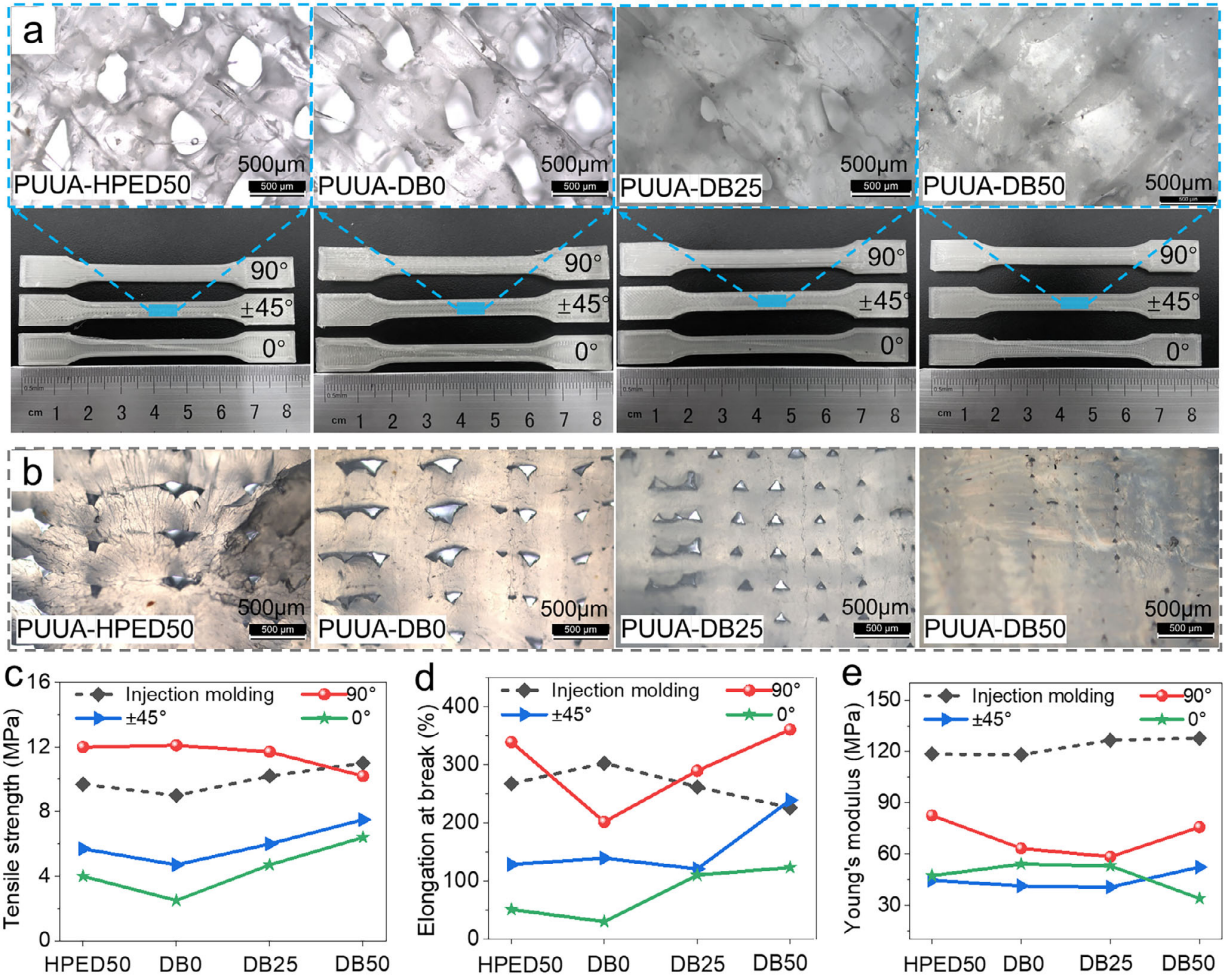
Printability of PUUA‐DB wires and enhancement of its interlayer multiple dynamic bonds for 4D printout. a) Polarized optical micrograph of PUUA‐DB printed samples. b) The cross‐section morphology of 90° printed samples after quenching brittle fracture in liquid nitrogen. c) Tensile strength, d) elongation at break, and e) Young's modulus of FDM‐printed PUUA‐DB parts with 0, ±45, and 90° raster angles. Scale bars: 500 µm.

The interfacial and cross‐section morphologies of printed PUUA‐DB samples were observed using POM (**Figure** **4**a,b). FDM‐printed samples with ±45° construction exhibited a distinct interleaved‐interconnected structure from the layer‐wise deposition. As the ED2003 content increased, the interlayer defects in printed PUUA‐DB50 samples with different raster angles were significantly decreased. Moreover, the interlayer defects in PUUA‐DB50 were notably fewer than in PUUA‐HPED50 (Figure S11, Supporting Information). This was attributed to the reversible destruction and construction of dynamic hydrogen bonds, as well as dynamic thiocarbamate and carbamate bonds, which facilitated the topological rearrangement in polymer networks. This, in turn, promoted enhanced intermolecular diffusion and entanglement between printing layers, reducing anisotropy in FDM‐printed samples.

As shown in Figure 4c–e and Figure S9b–d (Supporting Information), as the ED2003 content increased, the tensile strength, elongation at break, and printing accuracy of PUUA‐DBs improved. Notably, PUUA‐DB50 showed highest elongation at break, reaching 468.7±6.3%. The Y‐axis direction exhibited the largest deviation in FDM‐printed samples due to numerous interlayer defects and weaker interface adhesion, as evidenced by the lowest tensile strength observed in the 0° construction. Considering the thermal properties, mechanical properties, and 3D printability comprehensively, PUUA‐DB50 was selected to further study the interfacial enhancement mechanism to improve the shape memory and reconfiguration of 4D‐printed samples.

### 2.5 Shape Memory and Reconfiguration of 4D‐Printed PUUA‐DB50

**Figure 5:**
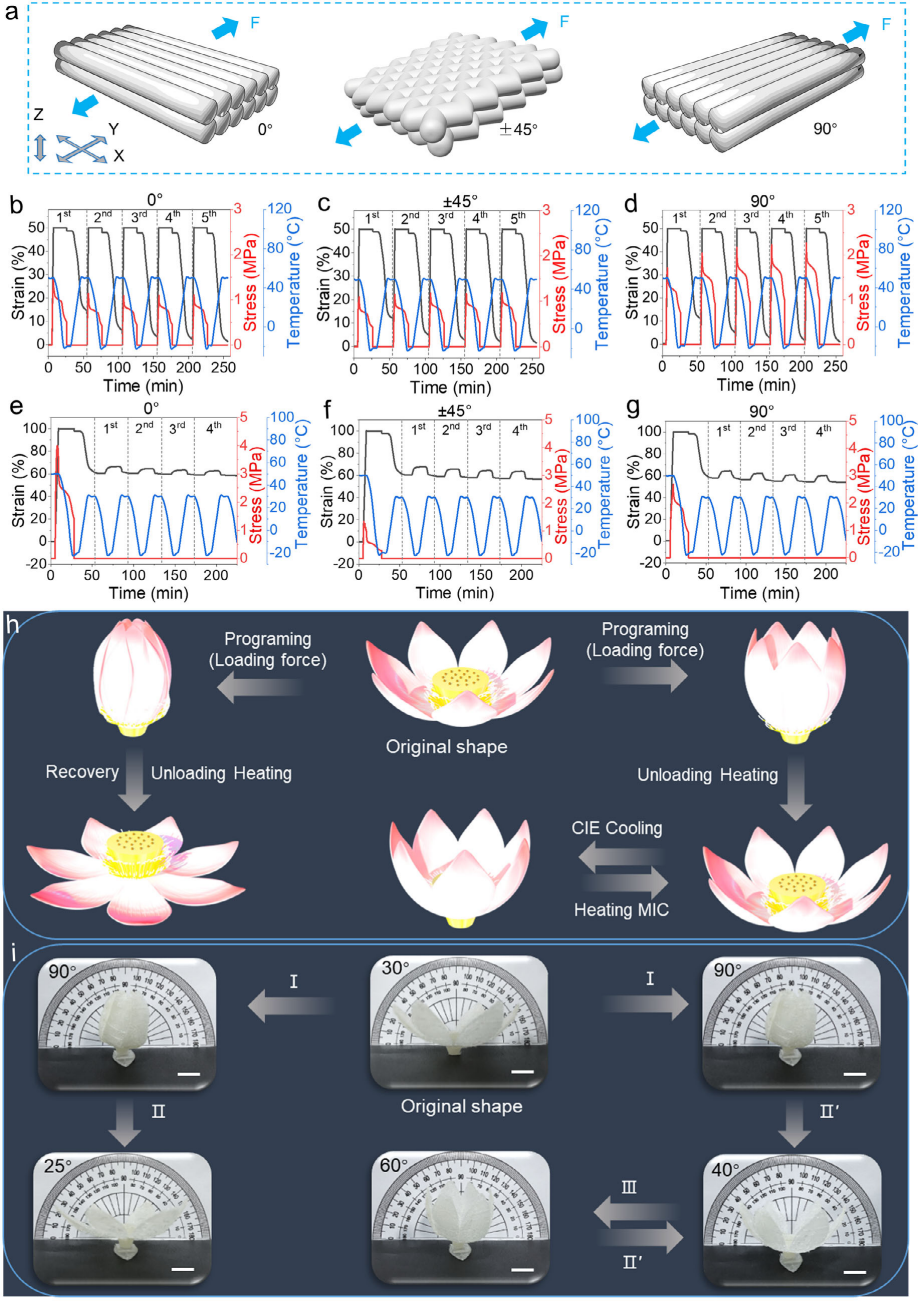
Influence of raster angle on the shape memory properties. a) The illustration of different raster angles and the blue arrow indicates the loading direction during the test. b–d) One‐way dual shape memory (*T*_low_ = −20 °C, *T*_high_ = 50 °C, *T*_high_ > *T*_switch_ > *T*_low_) and e–g) reversible two‐way shape memory (*T*_low_ = −20 °C, *T*_high_ = 30 °C) curves of PUUA‐DB50 printed films with 0, ±45, and 90° raster angles, respectively. h) Schematic diagram and i) macroscopic demonstration of one‐way dual shape memory and reversible 2W‐SME of PUUA‐DB50 printed lotus flowers (I: programing at 50 °C and fixing at −20 °C (The lotus petals were bent to a temporary shape at 50 °C, cooled to −20 °C, kept for 30 min, and then removed external force and temporary shape was fixed); II: heating to 50 °C; II*'*: heating to 30 °C; III: cooling to −20 °C). Scale bars, 10 mm.

To further investigate the impact of interfacial dynamic reversible bonds on enhancing shape memory performance, we characterized the one‐way dual shape memory, reversible two‐way shape memory, quadruple shape memory, and reconfiguration of 4D‐printed PUUA‐DB50 films with different filling directions (**Figure** **5**a). First, the crystallization and melting temperatures (*T*_c,S_ and *T*_m,S_) of soft segments in PUUA‐DB50 were used as responsive switches to achieve consecutive one‐way dual shape memory cycles (Figure 5b–d, data summarized in Tables S9–S11, Supporting Information). PA1212 and PETMP segments serve as physical and chemical crosslinking points, respectively, to enable permanent shape recovery. The average shape‐fixing ratio (*R*_f_ = 96.2%, 96.8%, and 97.1%) and the corresponding shape‐recovery ratio (*R*_r_ = 88.3%, 90.3%, and 90.9%) of 4D‐printed PUUA‐DB50 films gradually increased in the 0, ±45, and 90° filing directions.

Additionally, when the soft segments (PTMG and ED2003) formed a crystalline phase, the sample either elongated or contracted spontaneously. This behavior resulted from the crystallization and melting of PTMG and ED2003 domains along the orientated direction provided by the skeleton of PA1212 physically crosslinking network, and PETMP chemical crosslinking network. These processes are referred to as melting‐induced strain contraction (MIC) and crystallization‐induced strain elongation (CIE).^[^ ^38^ ^]^ As shown in Figure 5e‐g, the 4D‐printed PUUA‐DB50 films with 0, ±45, and 90° raster angles exhibited excellent and stable reversible two‐way shape memory when the temperature ranged from −20 to 30 °C. The average actuation ratio (*R*_act_) for sample with 0, ±45, and 90° rater angles were 4.6%, 6.9%, and 6.1% respectively, corresponding to the average recovery ratio (*R*_r, 2w_) of 100.0%, 106.6%, and 101.5% (Tables S12–S14, Supporting Information).

The results indicate that 4D‐printed PUUA‐DB50 films, regardless of raster angles, demonstrate excellent and stable one‐way dual as well as reversible two‐way shape memory. The low shape‐recovery ratios caused by interlayer defects, particularly in the 0° configuration, were mitigated. This improvement is attributed to multiple dynamic bonds that enhance intermolecular diffusion and entanglement between printing interlayers, thereby preserving the excellent mechanical property of polymer and ensuring consistent shape memory behavior across all filling directions. The 4D‐printed models of lotus flower (Figure S12 and Movie S1, Supporting Information), which display one‐way dual shape memory and reversible two‐way shape memory effects, serve as an excellent macroscopic demonstration (Figure 5h,i; Movie S2,S3, Supporting Information).

**Figure 6:**
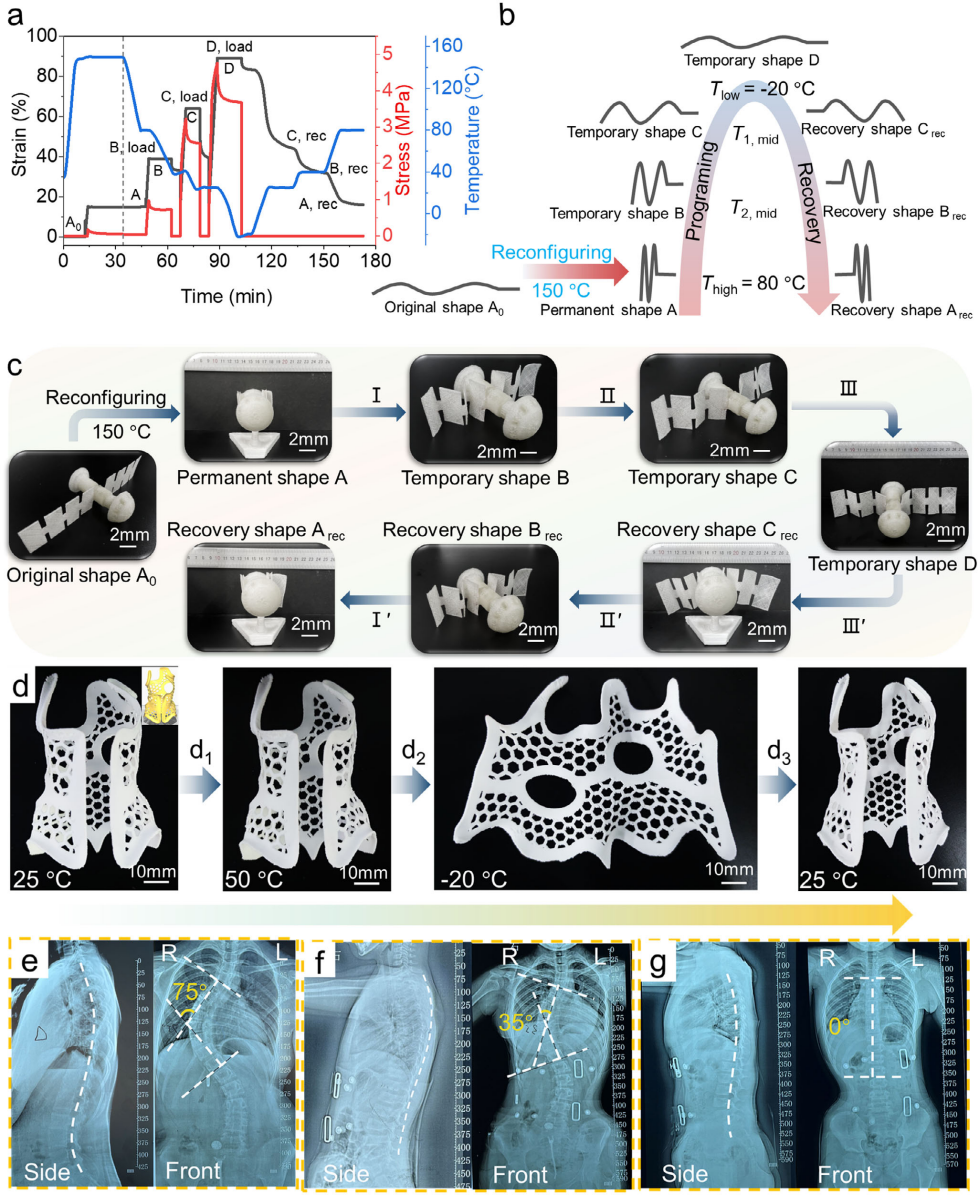
Reconfigurable 4D printing of multiple shape memory PUUA‐DB50 and its application in spinal orthoses. a) Reconfiguration (permanent deformation of 15% at 150 °C “A_0_→A”) and quadruple one‐way shape memory curve of printed PUUA‐DB50 film in the 90° configuration. b) Schematic diagram of the shape deformation, fixation, and recovery process of quadruple‐shape memory. c) Macroscopic demonstration of quadruple shape memory of PUUA‐DB50 printed aerospace (I: deforming at 80 °C and fixing at 40 °C; II: deforming at 40 °C and fixing at 25 °C; III: deforming at 25 °C and fixing at −20 °C; III*‘*: heating to 25 °C; II*’*: heating to 40 °C; I*'*: heating to 80 °C). d) Dual shape memory of the spinal orthosis (Inset: the digital file of spinal orthosis model) (d_1_: programing at 50 °C; d_2_: fixing at −20 °C (spinal orthosis was bent to a temporary shape of 180° angle at 50 °C, cooled to −20 °C, kept for 30 min, and then removed external force and temporary shape was fixed); d_3_: heating to 25 °C). e–g) Both side and frontal in‐brace radiographs of the volunteer wearing a 4D‐printed spinal orthosis at different stages of care (Cobb angle: 75°→35°→0°).

The temperature (*T*_min_ = 140 °C), the minimum relaxation time of PUUA‐DB50, was selected to activate the multiple dynamic covalent bonds (carbamate, thiocarbamate, and hydrogen bonds) (Figure 2g–i). As shown in **Figure** **6**a, the initial shape A_0_ of 4D‐printed PUUA‐DB50 film was first stretched to a strain of *ε*_0_ = 15% and placed at 150 °C (*T*_min_ < *T* < *T*_m, H_) for 20 min to achieve complete stress relaxation. The stress gradually decreased over time due to exchange reactions of dynamic covalent bonds, leading to a topological rearrangement of polymer chains and resulting in formation of a permanent shape A after 20 min.

Afterwards, the crystallization and melting temperatures of PTMG (*T*_c,S_ = −6.0 °C, *T*_m,S_ = 23.0 °C) and ED2003 (*T′*_c,S_ = −14.0 °C, *T′*_m,S_ = 30.0 °C) were used as shape memory switching temperatures *T*_1, switch_ and *T*_2, switch_, respectively, and the glass transition temperature (*T*_g_ = 62.8 °C) of the amorphous PA1212 phase served as *T*_3,switch_ (Figure 3b,c). Temporary shapes B and C were obtained sequentially upon cooling to 40, 25, and −20 °C, which resulted from the freezing of molecular chain movement in amorphous PA1212 domain and crystallization of PTMG and ED2003, respectively. During the heating process, PUUA‐DB50 transitioned from temporary shape D back to temporary shapes C, B, and A at 25, 40, and 80 °C, respectively. This transition occurred due to the entropy elasticity of the physical and chemical crosslinking network formed by PA1212 and PETMP, respectively. The recovery ratio *R*_r_ for temporary shapes B, C, and D was as high as 89.1%, 121.9%, and 90.0%, respectively, demonstrating excellent quadruple one‐way shape memory and reconfiguration (Table S15, Supporting Information). The 4D‐printed models of aerospace also demonstrated excellent macroscopic examples of one‐way quadruple shape memory effects and reconfiguration (Figure S13, Supporting Information; Figure 6b,c), consistent with the DMA results discussed earlier.

Furthermore, the spinal orthoses with multifunctional applications were fabricated using PUUA‐DB50 through 4D printing (Movie S4, Supporting Information), since PUUA‐DB50 possessed better shape memory and mechanical properties in the 90° configuration (Figure 5b–d). Specifically: (1) Strain capacity: this material achieved >90% recoverable strain (*R*_f_ = 97.1%, *R*_r_ = 90.9%) (Figure 5d; Table S11, Supporting Information), enabling for flexibility and conformability to the dynamic motions while maintaining structural integrity. (2) Transition temperature: actuation occurs within a biocompatible temperature range (*T*_high_ = 30 °C), which can be safely triggered by body heat and ensuring user comfort and practicality. (3) The mechanical properties are slightly lower than those reported in the current literature but are sufficient to meet practical application requirements, especially for the Shore A hardness of orthosis devices (Table S16, Supporting Information).^[^ ^7^, ^41^ ^]^ In particular, while enabling the realization of smart wearable orthotics, this material also inherently has a low bibulous rate and excellent wear resistance (Figure S14, Supporting Information and discussion), which thereby greatly enhanced the practicality and durability of spinal orthosis. The spine orthopedic scheme of 4D‐printed spinal orthosis and printing model was shown in Figure 6d and Figure S15 (Supporting Information), respectively. 4D‐printed spinal orthosis pattern to be fixed into temporary 1D shape at low temperature (−20 °C). Upon in‐brace spinal orthosis, the body temperature (>25 °C) triggers shape recovery of the 1D shape to its original 2D pattern, which could fine‐tune spinal orthosis shape to fit body structure conformally, providing flexibility and rigidity to support body movement and spine orthosis. The abnormal spine curvature of scoliosis was effectively alleviated by the treatment with 4D‐printed spinal orthosis at different stages of care, as evidenced by the gradual reduction in Cobb angle (Figure 6e–g). This novel PUUA‐DB material, synthesized through a cost‐effective approach, not only broadens 4D printable smart materials and alleviates mechanical anisotropy within 4D printouts, but also has potential in the fields of soft robots and the biomedical field.

## 3 Conclusion

We successfully prepared a series of interfacial reinforced shape memory PUUA‐DBs using block copolymerization in twin screw extruder through reactive extrusion. By introducing CANs into shape memory PUUA‐DB wires and utilizing the combined effects of dynamic thiocarbamate, carbamate, and multi‐strength hydrogen‐bonding interactions, we enhanced the interlayer adhesion. This improvement led to better mechanical properties in the 4D‐printed objects and reduced mechanical anisotropy. Notably, the 4D‐printed PUUA‐DB50 displayed excellent mechanical properties, including a tensile strength of 10.2 MPa, an elongation at break of 361.0%, and a Young's modulus of 75.7 MPa. 4D‐printed objects in various raster angles showed impressive one‐way dual shape memory (*R*_f_ = 97%, *R*_r_ = 91%), reversible two‐way shape memory (*R*_r,2w_ = 6.9%, *R*_act_ = 106.6%), quadruple shape memory (*R*_C,rec_ = 90.0%, *R*_B,rec_ = 121.9%, *R*_A,rec_ = 89.1%), demonstrating outstanding macroscopic shape memory and reconfiguration. Our experimental results illustrate that 4D‐printed spinal orthosis pattern using our synthesized polymer can fit human body structure conformally by changing from temporary 1D shape to its original 2D shape via shape memory effect only triggered by the body temperature. This novel PUUA‐DB elastomer not only expands range of 4D printable smart materials and reduces mechanical anisotropy, but also holds potential applications in soft robotics and the biomedical field.

## 4 Experimental Section

### Materials

Polytetramethylene ether glycol (PTMG, *M*_n_ = ≈2000) and dibutyltin dilaurate (DBTDL, 95%) were purchased from Aladdin Industrial Corp., China. Polyether amine (ED2003, *M*_n_ = ≈2000) was obtained from Huntsman International LLC. 4, 4′‐methylenebis (phenyl isocyanate) (MDI) was bought from Bayer, DEU. Pentaerythritol terakis (3‐mercaptopropionate) (PETMP, 90%) and *N*,*N*,*N'*,*N'*‐tetrakis (2‐hydroxypropyl) ethylenediamine (HPED, 98%) were purchased from Sigma‐Aldrich, USA. Dodecanedioic acid and 1,12‐dodecanediamine were purchased from Henan Junheng Industrial Group Co., Ltd., China. PTMG and ED2003 were dried at 120 °C under vacuum (−0.1 MPa) for 2 h to remove water, and other chemicals were used without any further purification. Polyamide 1212 oligomer powders (PA1212, *M*_n_ = ≈1200, PDI = 1.18) (Figure S16, Supporting Information) were synthesized according to the method which was reported in our previous work.^[^ ^38^ ^]^

### Synthesis of Isocyanate‐Terminated Polyurethane Prepolymer (OPU)

First, MDI (40.0 g, 104.6 mmol) and PTMG (26.2 g, 20.0 mmol) were added into the three‐necked flask equipped with mechanical stirrer, nitrogen inlet, and constant pressure funnel. The mixture was slowly heated to 80 °C and kept isothermally for 1.5 h in nitrogen flow at a stirring speed of 160 r min^−1^. Then, PETMP (3.0 g, 6.1 mmol) and DBTDL were added to react for 1 h. Finally, isocyanate‐terminated OPU prepolymer was obtained (Figure 1a). Specifically, the molar ratio of crosslinking agent (PETMP or HPED) in all samples was 3 mol%, and the content of DBTDL was 0.9 wt.%, which was determined in our previous work.^[^ ^39^ ^]^

### Synthesis of Micro‐Crosslinked Poly(Urethane‐Urea‐Amide) Elastomer (PUUA‐DB)

A series of PUUA‐DB elastomers with different ED2003 contents were prepared. First, PA1212 powder and ED2003 were mixed in a vacuum mixer. Then the premix and OPU were added into a twin‐screw extruder (Nanjing Giant SHJ‐36, China). Finally, PUUA‐DB elastomers were prepared by reactive extrusion method, followed by granulation and drying. The amino groups of PA1212 and ED2003 reacted with the isocyanate groups of OPU at a molar ratio of 1: 1. The temperature of twin‐screw extruder from the first section to the seventh section and die mouth were 155, 160, 165, 175, 185, 185, 185, and 180 °C, respectively, and the screw speed was 8 r min^−1^. The screw had a diameter of 36 mm and a length‐to‐diameter ratio of 10: 1. The PUUA‐DBs with different molar ratios of ED2003 were denoted as PUUA‐DB‐a, where “a” represents the molar ratio of ED2003 in soft segment. PUUA‐HPED50 was the contrast experiment of PUUA‐DB50, differing only in the substitution of PETMP with HPED (Table S2, Supporting Information). Additionally, the test samples for shape memory performance were all prepared using FDM printing.

### Preparation of 3D Printing Wires

The granular materials of PUUA‐DB were fed into a single screw extruder (Labtech LE25‐30C, USA) equipped with 2 mm mold for melting extrusion and hot‐stretching by pulling rollers into 1.75 ± 0.05 mm diameter wire. The temperature of single‐screw extruder from the first section to the fourth section and die mouth were 160, 170, 180, 190, and 165 °C, respectively, and the screw speed was 4 r min^−1^. 3D Printing methods were provided in the Supporting Information.

### Characterization: Chemical Structure

Fourier transform infrared spectroscopy (FTIR) was measured using a PerkinElmer Spectrum Two Spectrometer (USA) in ATR mode, with 64 scans at a resolution of 4 cm^−1^. The in situ variable temperature FTIR (VT‐FTIR) spectra were investigated by TR mode using the same spectrometer to detect the dynamic reversibility of hydrogen bonds. The samples were characterized at varying temperatures with KBr as the reference background, using 32 scans at a resolution of 1 cm^−1^ and a heating rate of 3 °C min^−1^.

### Characterization: Thermal Properties

Differential scanning calorimeter (DSC) (DSC214, Germany) was used to characterize the melting and crystallization behavior of PUUA‐DB. All samples were heated to 200 °C at a heating rate of 10 °C min^−1^ and isothermally incubated for 2 min to eliminate the effects of thermal history. The samples were cooled to −40 °C and then reheated to 200 °C at a rate of 10 °C min^−1^ under a nitrogen atmosphere.

### Characterization: Crystal Structure

The crystal structure was characterized using a high‐resolution X‐ray diffractometer SMARTLAB XRD (Japan) with the Cu *K*_α1_ (*λ* = 0.15 406 nm) as the radiation source, and the test was operated at 45 kV and 200 mA. The X‐ray Diffraction (XRD) spectra were collected at an ambient temperature of 25.0 ± 0.5 °C, scanning from 2*θ* = 5 to 90° at a rate of 10° min^−1^. All the samples were hot‐pressed into films of 1 mm thickness before testing.

### Characterization: Dynamic Mechanical Analysis

Dynamic mechanical analysis (DMA) was conducted using a TA Instruments Q800 (USA) in multifrequency‐strain mode under the following conditions: 1 Hz, 0.2% strain, and a cooling/heating rate of 3 °C min^−1^. Iso‐strain stress relaxation and shape memory experiments were conducted by the same DMA machine under strain‐rate mode. The characterizations of stress relaxation, dual‐shape memory, quadruple‐shape memory, and reversible two‐way shape memory performance were provided in the Supporting Information.

### Characterization: Mechanical Properties

Following the Chinese standard GB/T 1040.3‐2006, tensile tests were conducted on an MTS CMT4104 universal testing machine (USA) under crosshead speed of 50 mm min^−1^. The data of mechanical properties were averaged from five specimens (75 mm × 10 mm × 2 mm). The sample was fabricated using a miniature injection molding machine SZS‐20 (China), molten at 195 °C, and injected into stainless steel model with dumbbell‐shaped compression region.

Cyclic tensile tests were carried out using the same universal testing machine (200 N load cell) at a strain rate of 5 mm min^−1^ and a 20 mm initial grip distance, and the dimensions of samples were 40 mm × 6.3 mm × 0.5 mm.

### Characterization: Viscosity

The relative viscosity was performed according to GB/T12006.1‐1989. A 0.25 g sample was dissolved in 25 mL *m*‐cresol to prepare 1 g dL^−1^ polymer solution. Then, the flow time of *m*‐cresol and polymer solution was measured by Ubbelohde viscometer at 25 °C. Relative viscosity η_*r*_, specific viscosity η_*sp*_ and intrinsic viscosity [η] was obtained.

### Characterization: Melt Flow Rate

Following the Chinese standard GB/T 3682‐2000, the melt flow rate (*MFR*) of all samples was measured using a Melt Flow Indexer ZRZ1452 (USA) at 200 °C with a load of 2.16 kg.

### Characterization: Morphological Characterization

The interlayer and cross‐section morphology of FDM‐printed parts was observed using a polarized optical microscope (POM, Leica, DM2700P). The cross‐section of the 90° printed parts was obtained by quenching and embrittling in liquid nitrogen.

### Characterization: Radiograph

In‐brace radiographs were taken from the radiologist and orthopedic surgeon using an EOS radiography system (EOS Imaging S.A., Paris, France).

## Conflict of Interest

The authors declare no conflict of interest.

## Supporting information



Supporting Information

Supplemental Movie 1

Supplemental Movie 2

Supplemental Movie 3

Supplemental Movie 4

Supplemental Movie 5

## Data Availability

The data that support the findings of this study are available from the corresponding author upon reasonable request.
