# Supplementary material for: Dynamic Bonds Reinforced Polyamide Elastomer for Biomedical Orthosis
Source: Adv Sci (Weinh). 2025 May 20;12(30):e04395. doi: 10.1002/advs.202504395 (PMC12376624; doi:10.1002/advs.202504395)
Supplement: Supplementary file 1 — Supporting Information [file ADVS-12-e04395-s004.docx]

Supporting Information for

Dynamic Bonds Reinforced Polyamide Elastomer for Biomedical Orthosis

Zhen Li, Peiyao Yan, Hao Wang, Yuancheng Zhang*, Junhua Kong, Wei Zhao, Xin Li, Xiaomeng Zhang, Zhe Cui, Peng Fu*, Xinchang Pang, Minying Liu & Chaobin He*

Z. Li, Y. Zhang, W. Zhao, X. Li, X. Zhang, Z. Cui, P. Fu, X. Pang, M. Liu

School of Materials Science and Engineering, Zhengzhou University, Zhengzhou 450001, China.

E-mail: [yuanchengzh@zzu.edu.cn](mailto:yuanchengzh@zzu.edu.cn); [fupeng@zzu.edu.cn](mailto:fupeng@zzu.edu.cn)

Z. Li, P. Yan, H. Wang, C. He

Department of Materials Science and Engineering, National University of Singapore, Singapore 117575, Singapore.

E-mail: [msehc@nus.edu.sg](mailto:msehc@nus.edu.sg)

Y. Zhang, W. Zhao, X. Li, X. Zhang, Z. Cui, P. Fu, X. Pang, M. Liu

Zhengzhou University Industrial Technology Research Institute Co., Ltd, Zhengzhou 450001, China

Y. Zhang, W. Zhao, X. Li, X. Zhang, Z. Cui, P. Fu, X. Pang, M. Liu

Henan Key Laboratory of Advanced Nylon Materials and Application, Zhengzhou University, Zhengzhou 450052, China

Y. Zhang

Henan Tuoren Medical Device Co. Ltd, Xinxiang 453400, China

J. Kong, C. He

Institute of Materials Research and Engineering, Agency for Science Technology and Research (A^∗^STAR), Singapore 138634, Singapore

**Supplementary Notes: Tables**

**Table S1.** Comparison of the mechanical and shape memory properties of different FDM-printed polymers.

| Methods | Dynamic bonds | Additive manufacturing | Years | References |
| --- | --- | --- | --- | --- |
| Physical-assisted extrinsic methods | (Ionizing radiation) | FDM 3D printing | 2014 | [5] |
|  | (Post-heating) | FDM 3D printing | 2020 | [6] |
|  | (Infrared irradiation) | FDM 3D printing | 2021 | [7] |
|  | (Proton radiation) | FDM 3D printing | 2021 | [8] |
| Chemically designed intrinsic methods | (Surface segregating additive) | FDM 3D printing | 2019 | [10] |
|  | Dynamic Diels–Alder bonds | FDM 3D printing | 2017 | [14] |
|  | Dynamic urea bonds | FDM 3D printing | 2022 | [16] |
|  | Dynamic hindered urea and hierarchical hydrogen bonds | DLP 3D printing | 2024 | [17] |
|  | Multiple dynamic covalent bonds (carbamate, thiocarbamate) and hierarchical hydrogen bonds | FDM 4D printing | Our work | |

So far, various 4D printing techniques have been developed, including fused deposition modeling (FDM), direct ink writing, stereolithography, digital light processing and selective laser sintering.^[1]^ FDM, which creates 3D objects by building up layers of material, is the most widely used because its low-cost and easy to operate.^[2]^ Polymers are better suited for FDM than metals or ceramics due to their molecular structure and printability. Using polymers for 4D printing has opened up new applications in the biomedical field because it can construct intricate biological structures with high precision and efficiency, functionalizing personalized, regenerative and accessible treatments.^[3]^ Nevertheless, FDM printing still faces issues such as poor interlayer adhesion and mechanical properties, unstable shape memory properties. Traditional extrinsic methods, such as ionizing radiation,^[4]^ post-heating,^[5]^ infrared irradiation^[6]^ and proton radiation,^[7]^ can improve layer bonding but significantly increase manufacturing costs and reduce dimensional accuracy.^[8]^ For intrinsic approaches is the introduction of dynamic bonds(e.g. carbamate bond,^[9]^ thiocarbamate bond,^[10]^ disulfide bonds,^[11]^ Diels-Alder bonds,^[12]^ hydrogen bonds,^[13]^ metal coordination bonds,^[14]^ et al) in FDM-printed polymers.^[15]^

Poly(urethane-urea-amide) (PUUA) elastomer offers adjustable viscosity, a wide temperature memory range, excellent wear resistance, thermal stability, and superior fatigue resistance compared to traditional materials like polyurethane and polylactic acid.^[16]^ In our previous work, we developed a shape memory PUUA through reactive extrusion, an efficient and continuous method for synthesizing polymers.^[17]^ This material can be considered a novel, high-performance SMP suitable for 4D printing.^[18]^ Building on this, we propose that combining reactive extrusion with 4D printing could provide a more cost-effective and efficient approach to manufacturing 4D-printed SMPs. Additionally, multiple dynamic bonds were introduced into the PUUA to enhance the mechanical and shape memory properties of the printed structures.

**Table S2.** Feeding molar ratio of different components during the preparation of PUUA-DBs.

| Samples | MDI | PTMG | PETMP | HPED | ED2003 | OPA |
| --- | --- | --- | --- | --- | --- | --- |
| PUUA-HPED50 | 53 | 11 | 0 | 3 | 11 | 22 |
| PUUA-DB0 | 53 | 22 | 3 | 0 | 0 | 22 |
| PUUA-DB25 | 53 | 16.5 | 3 | 0 | 5.5 | 22 |
| PUUA-DB50 | 53 | 11 | 3 | 0 | 11 | 22 |

* The unit of all data in the table is mol%.

**Table S3.** Parameters for 3D printing used in FDM printer.

| Parameters | Value |
| --- | --- |
| Nozzle size (mm) | 0.4 |
| Infill density (%) | 100 |
| Printing speed (mm∙s^−1^) | 40 |
| Nozzle temperature (°C) | 200 |
| Layer height (mm) | 0.1 |
| Printing plate temperature(°C) | 60 |

**Table S4.** Assignment of infrared absorption peaks of PUUA-DBs.

| Wave number (cm^−1^) | The attribution of peaks |
| --- | --- |
| 3304 | N-H (Urea) |
| 2569 | S-H (Thiol) |
| 2264 | N=C=O |
| 1730 | C=O (Carbamate) |
| 1640-1630 | C=O (Urea, Amide Ⅰ band) |
| 1545-1535 | N-H (Amide Ⅱ band) |
| 1223 | C-N, C=O (Amide Ⅲ band) |
| 1105 | C-O-C |
| 721 | C-H (methylene) |
| 682 | C=O (Amide Ⅴ band) |
| 579 | N-H (Amide Ⅳ band) |

**Table S5.** Summary of the assignment of the deconvoluted subpeaks in the FTIR C=O absorption bands for PUUA-DBs.

| Assignment | | | Wavenumber (cm^−1^) | | | | Fitted Peak Area (%) | | | |
| --- | --- | --- | --- | --- | --- | --- | --- | --- | --- | --- |
|  |  |  | HPED50 | E0 | E25 | E50 | HPED50 | E0 | E25 | E50 |
| Carbamate (C=O) | Free | Ⅰ | 1728.6 | 1726.4 | 1727.4 | 1727.9 | 3.4 | 0.9 | 1.7 | 2.5 |
|  | H-bonded (ordered) | Ⅱ | 1705.8 | 1687.2 | 1691.6 | 1692.5 | 0.5 | 6.2 | 6.5 | 5.5 |
| Urea (C=O) | Free | Ⅲ | 1666 | 1659.5 | 1663.3 | 1666.1 | 5.5 | 15.2 | 8.8 | 7.1 |
|  | H-bonded (disordered) | Ⅳ | 1637 | 1635.5 | 1636.6 | 1639.4 | 61.8 | 52.7 | 54.2 | 51.1 |
|  | H-bonded (ordered) | Ⅴ | 1604.8 | 1612.2 | 1611.0 | 1617.7 | 9.4 | 1.1 | 5.1 | 9.1 |
| Amide (C=O) | H-bonded (ordered) | Ⅵ | 1597.3 | 1598.6 | 1597.8 | 1598.2 | 19.5 | 23.9 | 23.7 | 24.6 |
| Total degree of H-bonded | | | | | | | 91.1 | 83.9 | 89.5 | 90.4 |

**Table S6.** Thermal performance of PUUA-DBs.

| Samples | *T*_d, 5%_ ^a)^  (°C) | *T*_d, mid_ ^b)^  (°C) | *T*_d, max_ ^b)^  (°C) | Residual mass  (%) |
| --- | --- | --- | --- | --- |
| PUUA-HPED50 | 300.6 | 319.5 | 412.2 | 1.7 |
| PUUA-DB0 | 286.1 | 324.9 | 415.0 | 2.5 |
| PUUA-DB25 | 293.0 | 326.4 | 415.3 | 1.6 |
| PUUA-DB50 | 293.5 | 324.9 | 409.6 | 1.0 |

^a)^ *T*_d, 5%_ is the thermal degradation temperature for 5% mass loss.

^b)^ *T*_d, mid_ is and *T*_d, max_ are the maximum thermal degradation temperatures corresponding to the first and second peak temperatures in the DTG curve, respectively.

**Discussion:** As shown in Supplementary **Table 6**, the first stage (250 °C - 380 °C) mainly involves the thermal degradation (*T*_d, mid_) of less thermally stable carbamate and urea bonds, while the second stage (380 °C - 500 °C) primarily involves the thermal degradation (*T*_d, max_) of relatively stable C-N bonds in amide groups.

**Table S7.** The crystallization temperature (*T*_c_), melting temperature (*T*_m_) and glass transition temperature (*T*_g_) of PUUA-DB filaments.

| Samples | *T*_c, S_ ^a)^ | *T*_m, S_ ^a)^ | *T*_c, H_ ^b)^ | *T*_m, H_ ^b)^ | *T*_β_ ^c)^ | *T*_g_ ^d)^ |
| --- | --- | --- | --- | --- | --- | --- |
| PUUA-HPED50 | -12.0/2.0 | 26.0 | 90.0 | 154.0 | -42.9 | 61.6 |
| PUUA-DB0 | -3.7 | 23.3 | - | - | -45.4 | 52.7 |
| PUUA-DB25 | -16.0 | 16.0/25.0 | - | - | -33.0 | 53.5 |
| PUUA-DB50 | -14.0/-6.0 | 23.0/30.0 | 108.0 | 152.0 | -32.4 | 62.8 |

* The unit of all data in the table is °C.

^a)^ *T*_c, S_ and *T*_m, S_ are the crystallization and melting temperature of PTMG soft segments in PUUA-DBs, respectively.

^b)^ *T*_c, S_ and *T*_m, S_ are the crystallization and melting temperature of PA1212 hard segments in PUUA-DBs, respectively.

^c)^ *T*_β_ is the β transition temperature of PTMG segments in PUUA-DBs.

^d)^ *T*_g_ is the glass transition temperature of PA1212 amorphous phase in PUUA-DBs.

**Table S8.** Intrinsic viscosity $[\eta]$ and melt flow rate (*MFR*) of PUUA-DBs.

| Samples | *t* (s) | $\eta_{r}$ | $[\eta]$ | *MRC* (g min^−1^) |
| --- | --- | --- | --- | --- |
| PUUA-HPED50 | 521.15 | 2.61 | 1.14 | 19.92 |
| PUUA-DB0 | 534.53 | 2.68 | 1.20 | 18.54 |
| PUUA-DB25 | 516.47 | 2.60 | 1.12 | 19.51 |
| PUUA-DB50 | 513.55 | 2.54 | 1.11 | 20.07 |

**Table S9.** The *R*_f_ and *R*_r_ of dual-shape memory for 0° printed PUUA-DB50 sample.

| Raster angle | Cycles | PUUA-DB50 | | | | *R*_f_ | *R*_r_ |
| --- | --- | --- | --- | --- | --- | --- | --- |
|  |  | *ε*_0_ | *ε*_load_ | *ε*_unload_ | *ε*_r_ |  |  |
| 0° | 1 | 0 | 50 | 48.8 | 14.7 | 97.6 | 69.9 |
|  | 2 | 0 | 50 | 48.1 | 6.3 | 96.2 | 86.9 |
|  | 3 | 0 | 50 | 48.0 | 3.4 | 96.0 | 92.9 |
|  | 4 | 0 | 50 | 47.9 | 2.4 | 95.8 | 95.0 |
|  | 5 | 0 | 50 | 47.7 | 1.5 | 95.4 | 96.9 |
| *‾X*^a)^ | | | | | | 96.2 | 88.3 |
| *S* ^b)^ | | | | | | 0.8 | 11.0 |

* The unit of all data in the table is %.

^a)^*‾X* is the average value of PUUA-DB50 in the first five cycles.

^b)^ *S* is the standard deviation of PUUA-DB50 in the first five cycles.

**Table S10.** The *R*_f_ and *R*_r_ of dual-shape memory for ±45° printed PUUA-DB50 sample.

| Raster angles | Cycles | PUUA-DB50 | | | | *R*_f_ | *R*_r_ |
| --- | --- | --- | --- | --- | --- | --- | --- |
|  |  | *ε*_0_ | *ε*_load_ | *ε*_unload_ | *ε*_r_ |  |  |
| ±45° | 1 | 0 | 50 | 49.0 | 12.0 | 98.0 | 75.51 |
|  | 2 | 0 | 50 | 48.4 | 5.0 | 96.8 | 89.67 |
|  | 3 | 0 | 50 | 48.2 | 2.9 | 96.4 | 93.98 |
|  | 4 | 0 | 50 | 48.2 | 2.1 | 96.4 | 95.64 |
|  | 5 | 0 | 50 | 48.1 | 1.5 | 96.2 | 96.88 |
| *‾X*^a)^ | | | | | | 96.8 | 90.34 |
| *S* ^b)^ | | | | | | 0.7 | 8.7 |

* The unit of all data in the table is %.

^a)^*‾X* is the average value of PUUA-DB50 in the first five cycles.

^b)^ *S* is the standard deviation of PUUA-DB50 in the first five cycles.

**Table S11.** The *R*_f_ and *R*_r_ of dual-shape memory for 90° printed PUUA-DB50 sample.

| Raster angle | Cycles | PUUA-DB50 | | | | *R*_f_ | *R*_r_ |
| --- | --- | --- | --- | --- | --- | --- | --- |
|  |  | *ε*_0_ | *ε*_load_ | *ε*_unload_ | *ε*_r_ |  |  |
| 90° | 1 | 0 | 50 | 49.1 | 11.3 | 98.2 | 77.0 |
|  | 2 | 0 | 50 | 48.6 | 5.2 | 97.2 | 89.3 |
|  | 3 | 0 | 50 | 48.4 | 2.8 | 96.8 | 94.2 |
|  | 4 | 0 | 50 | 48.3 | 1.8 | 96.6 | 96.3 |
|  | 5 | 0 | 50 | 48.3 | 1.1 | 96.6 | 97.7 |
| *‾X*^a)^ | | | | | | 97.1 | 90.9 |
| *S* ^b)^ | | | | | | 0.7 | 8.4 |

* The unit of all data in the table is %.

^a)^*‾X* is the average value of PUUA-DB50 in the first five cycles.

^b)^ *S* is the standard deviation of PUUA-DB50 in the first five cycles.

**Table S12.** The reversible response strain (*ε*_2w_), actuation ratio (*R*_act_) and recovery ratio (*R*_r, 2w_) of reversible 2W-SME for 0° printed sample of PUUA-DB50 in the temperature range of −20 °C ~ 30 °C.

| Raster angle | PUUA-DB50 | | | | *ε*_2w_ | *R*_act_ | *R*_r, 2w_ |
| --- | --- | --- | --- | --- | --- | --- | --- |
|  | Cycles | *ε*_0_ | *ε*_low_ | *ε*_high_ |  |  |  |
| 0° | 1 | 60.6 | 66.5 | 61.2 | 5.9 | 6.0 | 89.8 |
|  | 2 | 60.4 | 64.7 | 60.3 | 4.3 | 4.4 | 102.3 |
|  | 3 | 59.7 | 63.5 | 59.5 | 3.8 | 3.9 | 105.3 |
|  | 4 | 59.0 | 62.8 | 58.9 | 3.8 | 3.9 | 102.6 |
| *‾X*^a)^ | | | | | 4.5 | 4.6 | 100.0 |
| *S* ^b)^ | | | | | 0.99 | 1.04 | 47.82 |

* The unit of all data in the table is %.

^a)^*‾X* is the average value of PUUA-DB50 in the first four cycles.

^b)^ *S* is the standard deviation of PUUA-DB50 in the first four cycles.

**Table S13.** The reversible response strain (*ε*_2w_), actuation ratio (*R*_act_) and recovery ratio (*R*_r, 2w_) of reversible 2W-SME for ±45° printed sample of PUUA-DB50 in the temperature range of −20 °C ~ 30 °C.

| Raster angles | PUUA-DB50 | | | | *ε*_2w_ | *R*_act_ | *R*_r, 2w_ |
| --- | --- | --- | --- | --- | --- | --- | --- |
|  | Cycles | *ε*_0_ | *ε*_low_ | *ε*_high_ |  |  |  |
| ±45° | 1 | 60.5 | 67.7 | 60.0 | 7.2 | 7.4 | 106.9 |
|  | 2 | 59.1 | 65.8 | 58.5 | 6.7 | 6.9 | 109.0 |
|  | 3 | 58.0 | 64.5 | 57.6 | 6.5 | 6.7 | 106.2 |
|  | 4 | 57.2 | 63.8 | 56.9 | 6.6 | 6.8 | 104.5 |
| *‾X*^a)^ | | | | | 6.8 | 6.9 | 106.6 |
| *S* ^b)^ | | | | | 0.10 | 0.10 | 3.36 |

* The unit of all data in the table is %.

^a)^*‾X* is the average value of PUUA-DB50 in the first four cycles.

^b)^ *S* is the standard deviation of PUUA-DB50 in the first four cycles.

**Table S14.** The reversible response strain (*ε*_2w_), actuation ratio (*R*_act_) and recovery ratio (*R*_r, 2w_) of reversible 2W-SME for 90° printed sample of PUUA-DB50 in the temperature range of −20 °C ~ 30 °C.

| Raster angle | PUUA-DB50 | | | | *ε*_2w_ | *R*_act_ | *R*_r, 2w_ |
| --- | --- | --- | --- | --- | --- | --- | --- |
|  | Cycles | *ε*_0_ | *ε*_low_ | *ε*_high_ |  |  |  |
| 90° | 1 | 58 | 64.5 | 57.5 | 6.5 | 6.7 | 107.7 |
|  | 2 | 56.4 | 62.3 | 56 | 5.9 | 6 | 106.8 |
|  | 3 | 55.2 | 60.9 | 55.1 | 5.7 | 5.8 | 101.8 |
|  | 4 | 54.5 | 60.2 | 54.4 | 5.7 | 5.8 | 101.8 |
| *‾X*^a)^ | | | | | 6 | 6.1 | 104.5 |
| *S* ^b)^ | | | | | 0.14 | 0.15 | 10.15 |

* The unit of all data in the table is %.

^a)^*‾X* is the average value of PUUA-DB50 in the first four cycles.

^b)^ *S* is the standard deviation of PUUA-DB50 in the first four cycles.

**Table S15.** The shape-fixing ratio (*R*_f_) and shape-recovery ratio (*R*_r_) of quadruple one-way shape memory for PUUA-DB50 printed film in the 90° configuration at different stages of one cycle.

| Stages | PUUA-DB50 | | | | | | | | | | *R*_f_ | *R*_r_ |
| --- | --- | --- | --- | --- | --- | --- | --- | --- | --- | --- | --- | --- |
|  | *ε*_A_ | *ε*_B_ | *ε*_C_ | *ε*_D_ | *ε*_B, unloa_ _d_ | *ε*_C, unload_ | *ε*_D, unloa_ _d_ | *ε*_A, r_ | *ε*_B, r_ | *ε*_C, r_ |  |  |
| A↔B | 0 | 25.0 | / | / | 19.2 | / | / | 2.1 | / | / | 76.8 | 89.1 |
| B↔C | / | 25.0 | 50.0 | / | / | 25.6 | / | / | 17.8 |  | 20.8 | 121.9 |
| C↔D | / | / | 50.0 | 75.0 | / | / | 69.3 | / | / | 30.0 | 88.5 | 90.0 |

* The unit of all data in the table is %.

**Table S16.** Shore D Hardness of PUUA-DBs.

| Samples | Shore D |
| --- | --- |
| PUUA-HPED50 | 36.5 |
| PUUA-DB0 | 35.2 |
| PUUA-DB25 | 36.7 |
| PUUA-DB50 | 36.8 |

**Supplementary Notes: Figures**


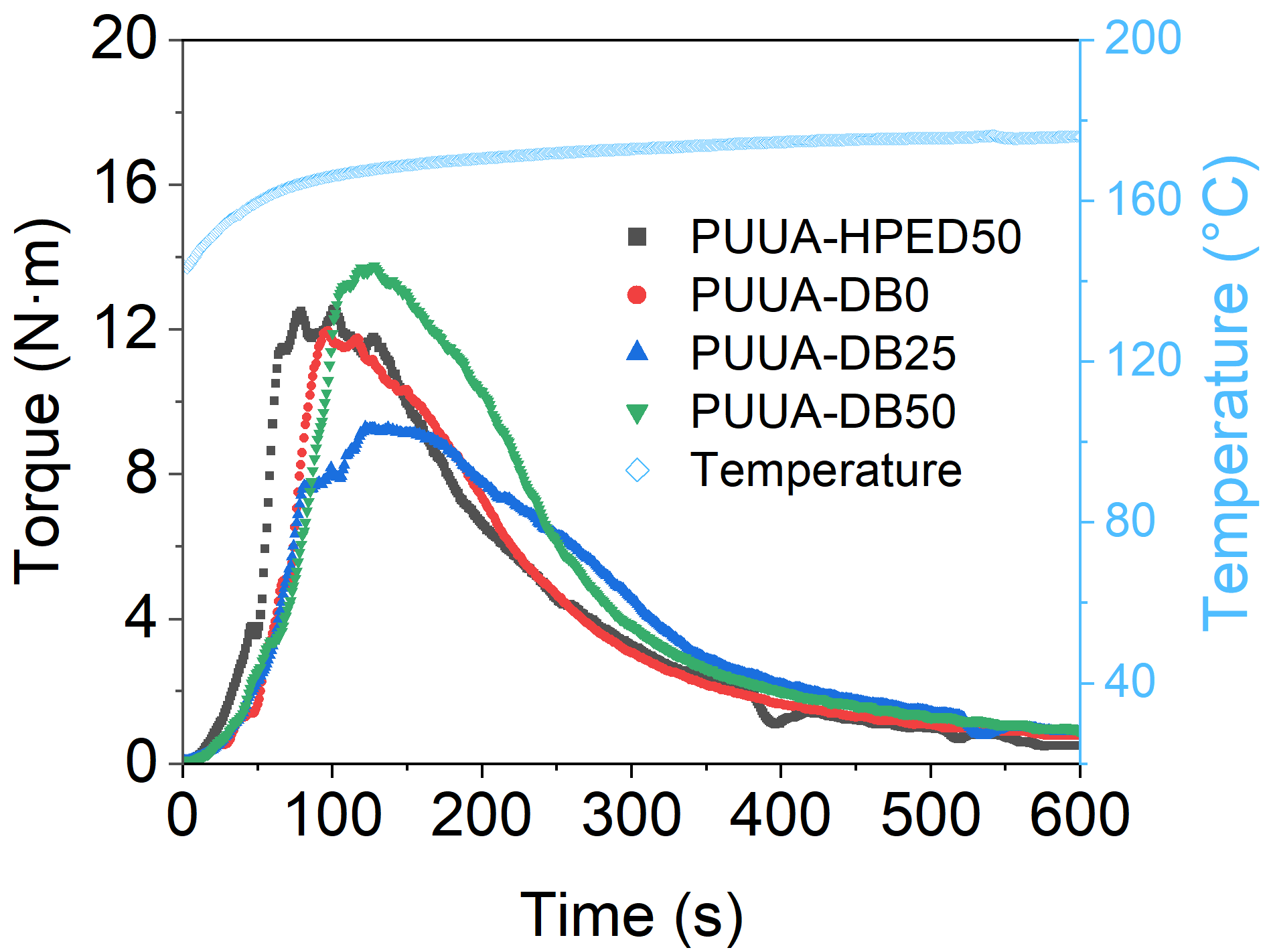


**Figure S1.** Torque and real-time temperature curves of PUUA-DBs.

**Discussion:**

Firstly, amino-terminated PA1212 and ED2003, as well as isocyanate-terminated OPU, were introduced into a vacuum mixer at an equimolar ratio and pre-mixed for 10 minutes. Subsequently, the premix was transferred to a torque rheometer, where it was reacted at 175 °C and 30 r min^-1^ for 10 minutes. Upon reaching equilibrium as indicated by the torque curve, uniform PUUA-DBs were successfully synthesized. As shown in **Figure S1**, all the samples have similar torque-time curves, which can be divided into three phases: The initial phase is the feeding stage, during which the torque remains relatively low due to the low viscosity of the premix. The second phase corresponds to the polymerization reaction stage. In this stage, as the PA1212 oligomer gradually melts, the collision probability of reactive groups (-NH_2_ and -NCO) increases, thereby accelerating the polymerization reaction rate. This leads to an increase in both the molecular weight and viscosity of the copolymer, resulting in a rapid rise in torque. The final phase is the homogenization stage. At this point, the polymerization reaction is essentially complete. The high viscosity of the polymer causes significant friction with the rotor, generating heat energy and inducing shear thinning. Consequently, the torque initially decreases before stabilizing.


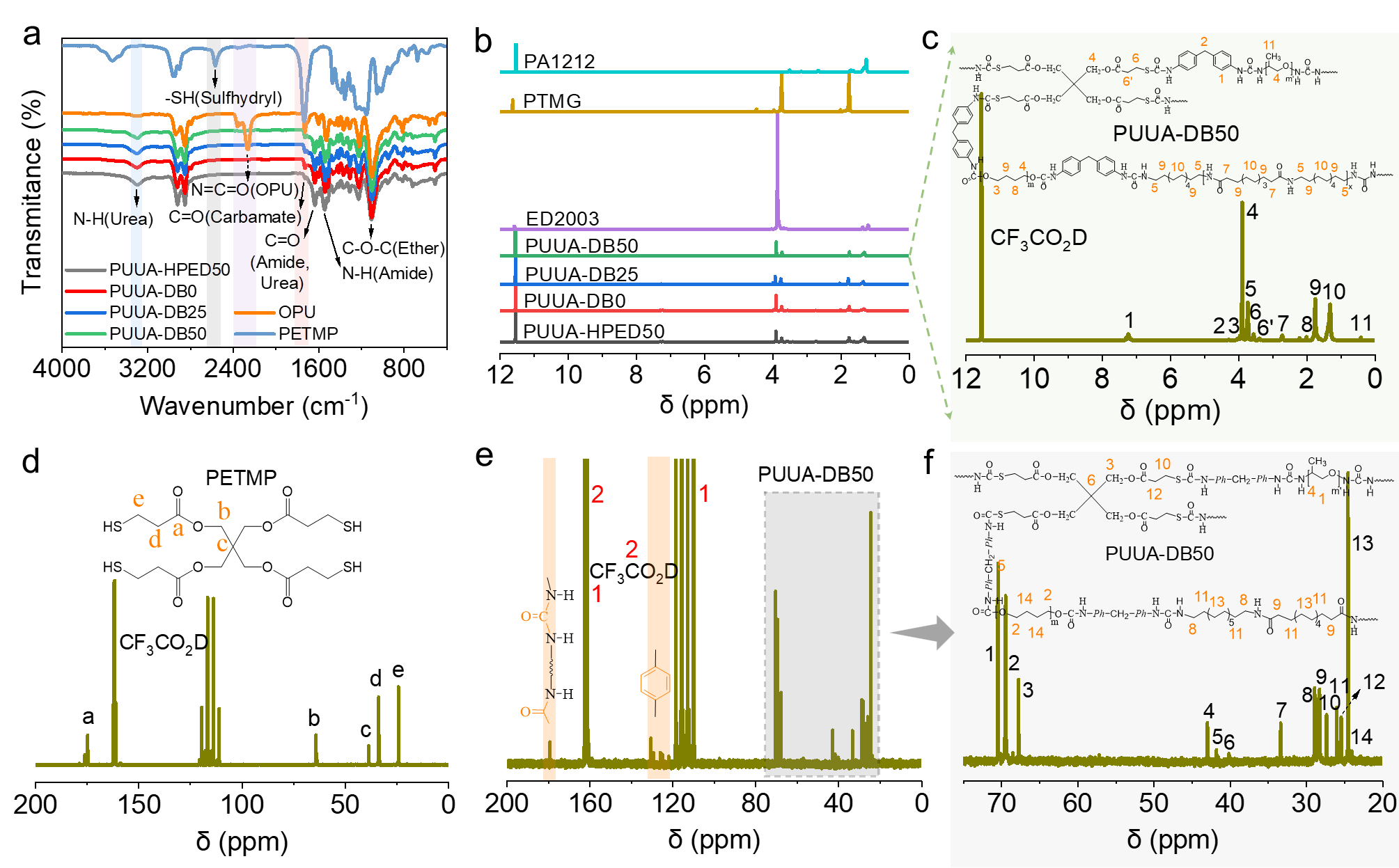


**Figure S2.** **Structure characterizations.** (**a**) FTIR spectra of PETMP, OPU and PUUA-DBs. (**b, c**) ^1^H NMR spectra of ED2003, PTMG, PA1212 and PUUA-DBs. The ^13^C NMR spectra of (**d**) PETMP and (**e, f**) PUUA-DB50.

**Discussion:**

FTIR analysis was carried out to characterize the chemical structure of OPU, PETMP and PUUA-DBs (**Figure S2**a). Assignment of infrared absorption peaks of PUUA-DBs was summarized in **Table S4**. The characteristic peaks of the -NCO groups at 2264 cm^−1^ and the -SH groups at 2569 cm^−1^ in PUUA-DBs both disappeared, while a new peak at 3304 cm^−1^ appeared, indicating N-H stretching of urea. This suggests that the -NCO groups reacted with -OH, -SH and -NH_2_ to form carbamate (−NHCOO−), thiocarbamate (−NHCOS−) and urea (−NHCONH−). Additionally, absorption peaks at 1105 cm^−1^ and 1544 cm^−1^ were attributed to the ether bonds (C-O-C) and N-H bending of amide Ⅱ bands, respectively, confirming the successful incorporation of PTMG and PA1212 segments. The absorption peak at 1635 cm^−1^ was attributed to the C=O stretching of amide Ⅰ and Ⅱ bands, as well as urea. The existence of urea was confirmed by correlating it with the characteristic N-H stretching vibration of the −NHCONH− groups at 3304 cm^−1^. Furthermore, to further analyze the C=O stretching vibration region and its associated hydrogen bonding interactions, peak deconvolution was performed. The corresponding results and discussion are presented in **Figure S3** and **Table S5**.

To further analyze the chemical structure of PUUA-DBs, ^1^H and ^13^C NMR spectroscopy was conducted and detailed information can be found in **Figure S2**b-f. ^1^H NMR spectra were shown in **Figure S2**b, c. Taking PUUA-DB50 as an example for analysis, the protons of the PETMP segments (3.9 ppm (4), 3.6 ppm (6)) correspond to the protons of methylene connected to ester bond in PETMP.^[18]^ The protons of PA1212 (3.7 ppm (5), 2.7 ppm (7), 1.8 ppm (9), 1.3 (10)) and PTMG (4.3 ppm (3), 3.9 ppm (4), 2.0 ppm (8), 1.8 ppm (9)) segments are consistent with the ^1^H NMR results (**Figure S2**b) of PA1212 and PTMG, respectively. Additionally, the chemical shift (δ) at 0.3 ppm (11) is attributed to the methyl protons in the ED2003 segments, corresponding to the chemical structure of PUUA-DB50. ^13^C NMR spectra were shown in **Figure S2**d-f. The signal at 179.3 ppm corresponds to the carbonyl carbon in both carbamate and urea, while the signals within the range of 130.6 ppm - 122.1 ppm represent the chemical shift of carbon in benzene. The chemical shifts at 70.5 ppm (1) and 43.0 ppm (4) originate from α-C and β-C of ether in ED2003 segments, respectively. Furthermore, the chemical shifts of PETMP segments (67.8 ppm (3), 40.2 ppm (6), 27.4 ppm (10), 25.4 (12)) are consistent with the ^13^C NMR results (**Figure S2**d) of PETMP (b, c, e, d). The signal peeks at 28.9, 28.3, 26.0 and 24.5 ppm corresponded to α-C (8, 9), β-C (11) and 3-10 carbon (13) connected to the N-H and C=O of amide bonds in PA1212 segments, confirming the successful synthesis of PUUA-DB elastomer.


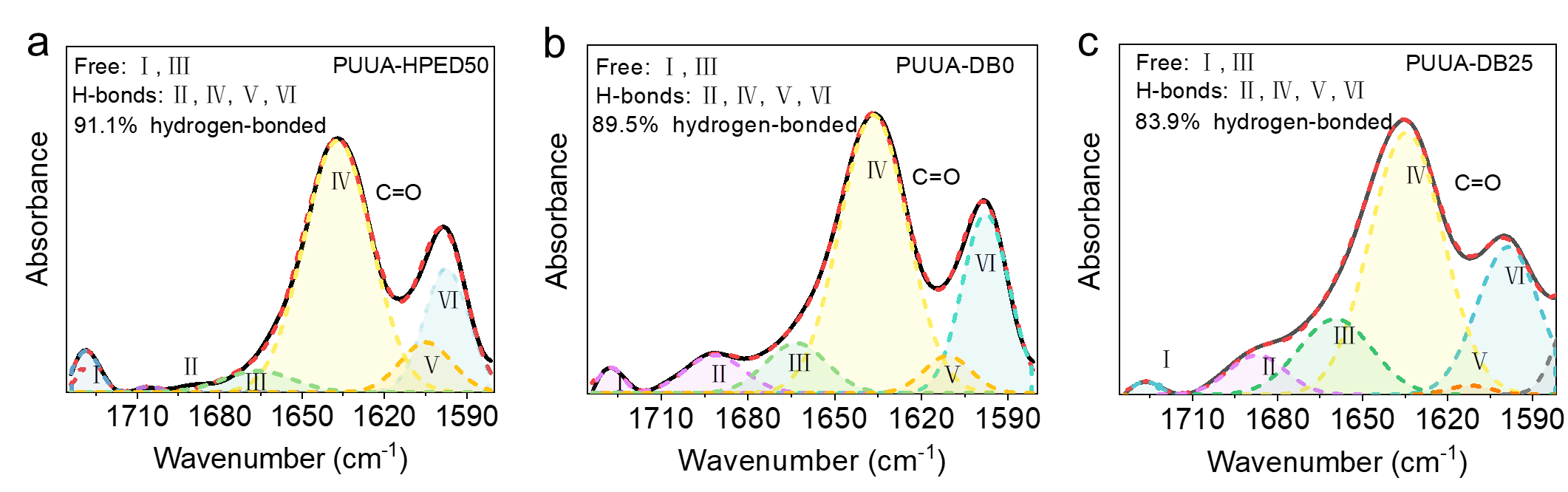


**Figure S3.** The deconvoluted subpeaks of FTIR spectra in the C=O stretching vibration region for (**a**) PUUA-HPED50, (**b**) PUUA-DB0 and (**c**) PUUA-DB25.

**Discussion:** The C=O absorption bands were deconvoluted into seven subpeaks based on the Gauss-Lorenz function. The deconvoluted subpeaks were assigned to the free and hydrogen-bonded C=O in the carbamate, urea and amide groups. The C=O stretching vibrations of the free, disordered and ordered hydrogen bonds were summarized in **Table S5**.


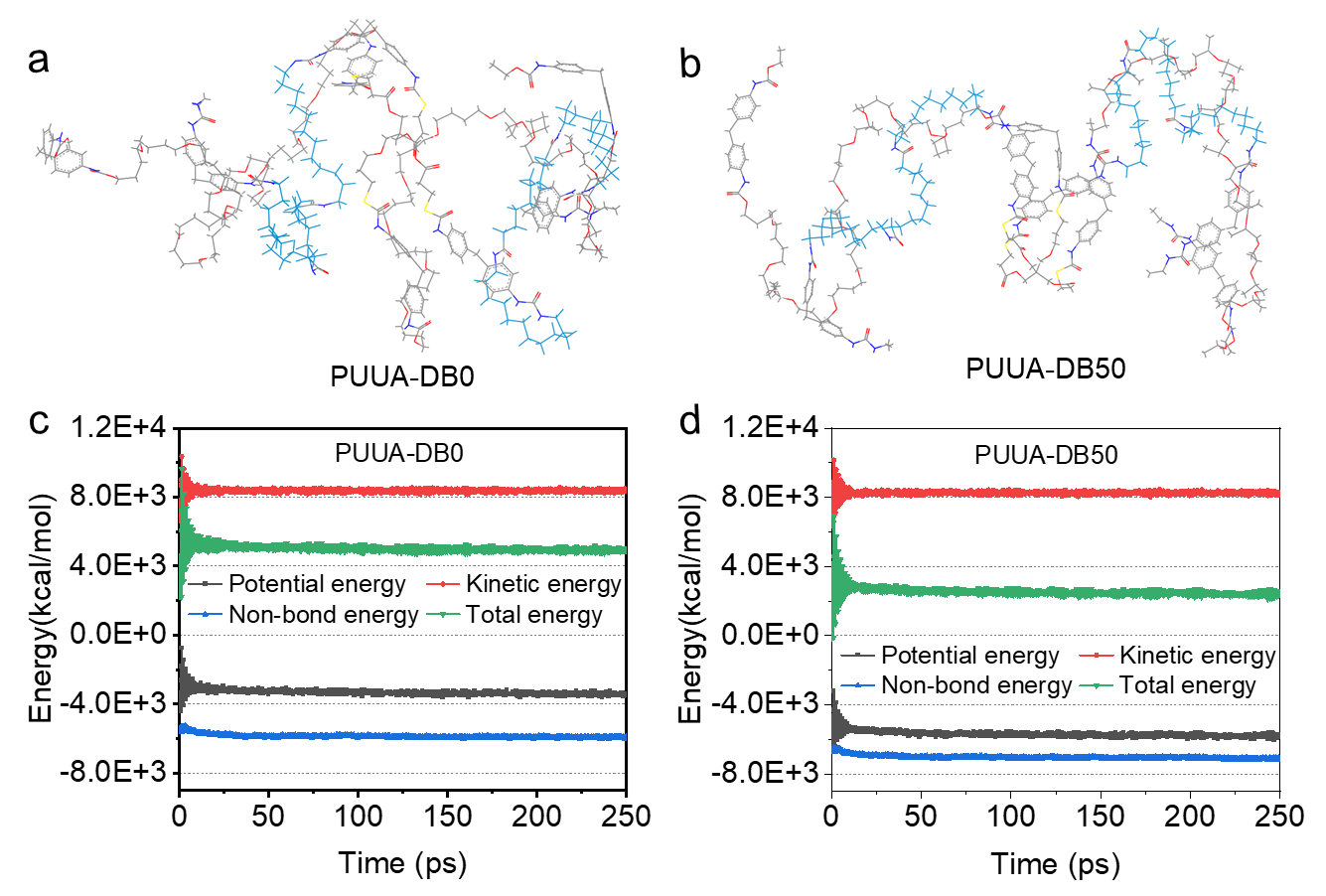


**Figure S4.** Optimized polymer chain of PUUA-DB0 (**a**) and PUUA-DB50 (**b**) by simulations. Forcite dynamics energies of PUUA-TU0 (**c**) and PUUA-TU3-H10 (**d**) in simulation cells after annealing.


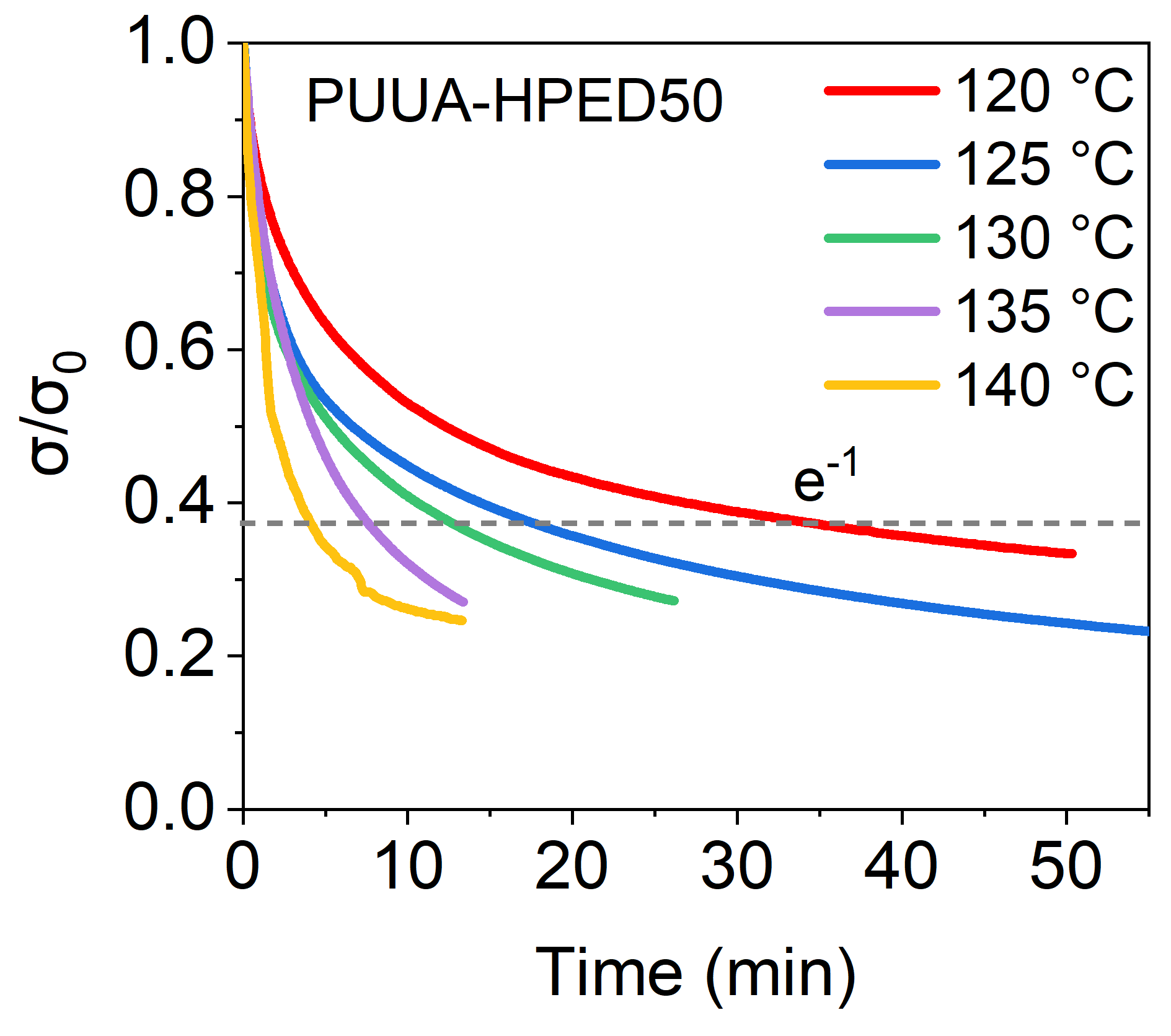


**Figure S5.** Normalized stress vs time curves of PUUA-HPED50.


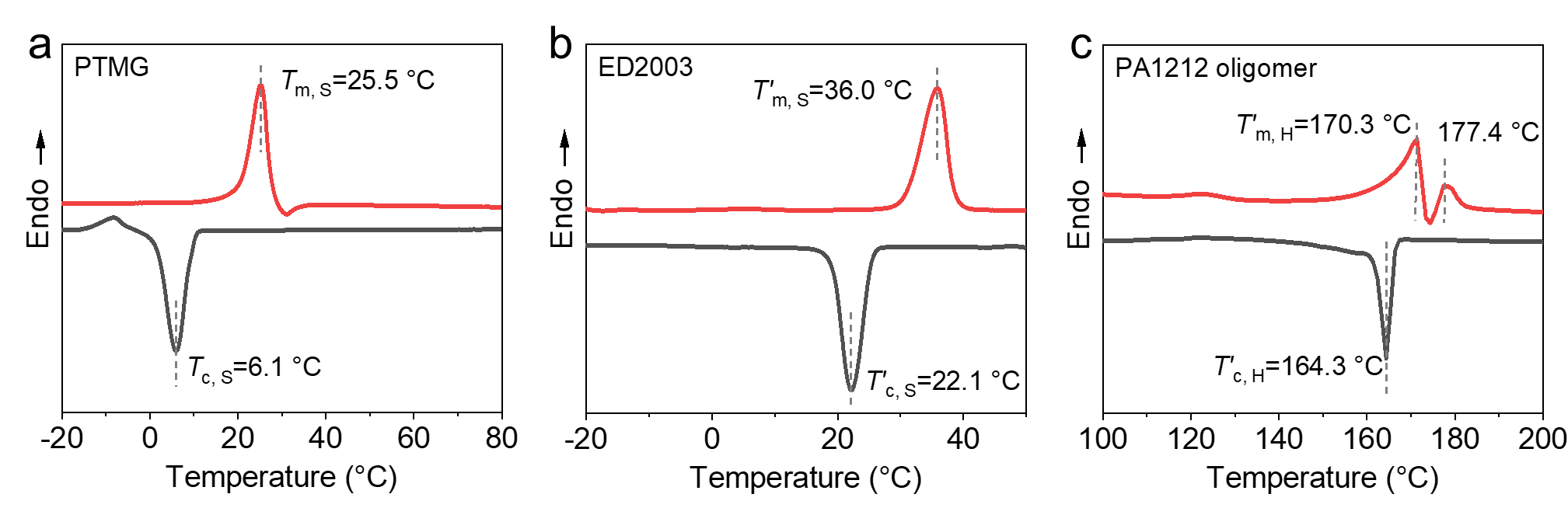


**Figure S6.** DSC cooling curves and secondary heating curves of (**a**) PTMG, (**b**) ED2003 and (**c**) PA1212 oligomer.


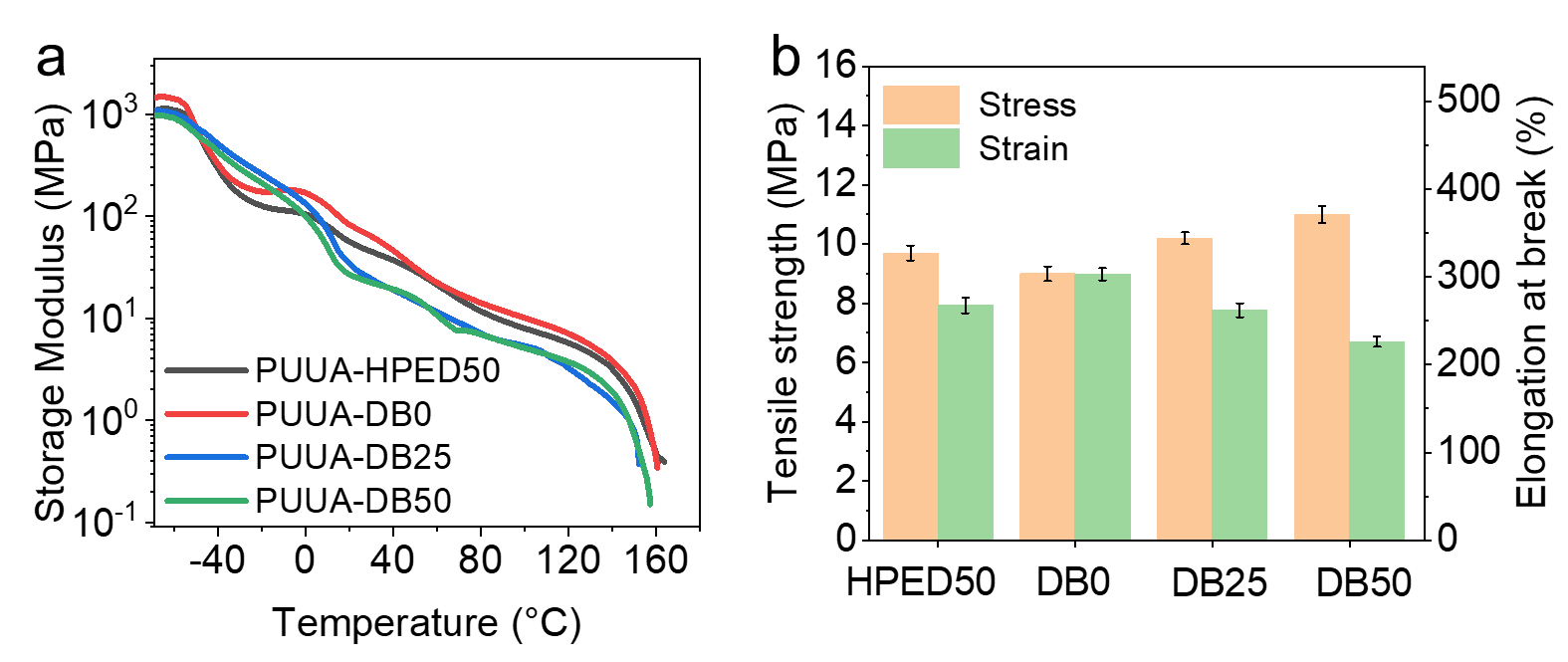


**Figure S7.** (**a**) Storage modulus (*E′*) of PUUA-DBs. (**b**) Strength and elongation at break of PUUA-DBs.

**Discussion:** As shown in **Figure S7**a, PUUA-DBs exhibited similar rheological curves. Among these, the β relaxation peak in the low temperature range (−60 °C ~ 0 °C) was associated with the synergy of two relaxations (**Figure 3**e): One relaxation corresponded relaxation transition (*T*_β_) of amorphous PTMG segments, while the other was the movement of amorphous PA1212 segments, including amide groups and methylene segments that do not participate in hydrogen bonding. In contrast, the α relaxation transition in the high temperature range (0 °C ~ 100 °C) indicated a transformation from crystalline phase to amorphous state in PA1212, corresponding the glass transition temperature (*T*_g_) of PA1212 amorphous phase.^[19]^


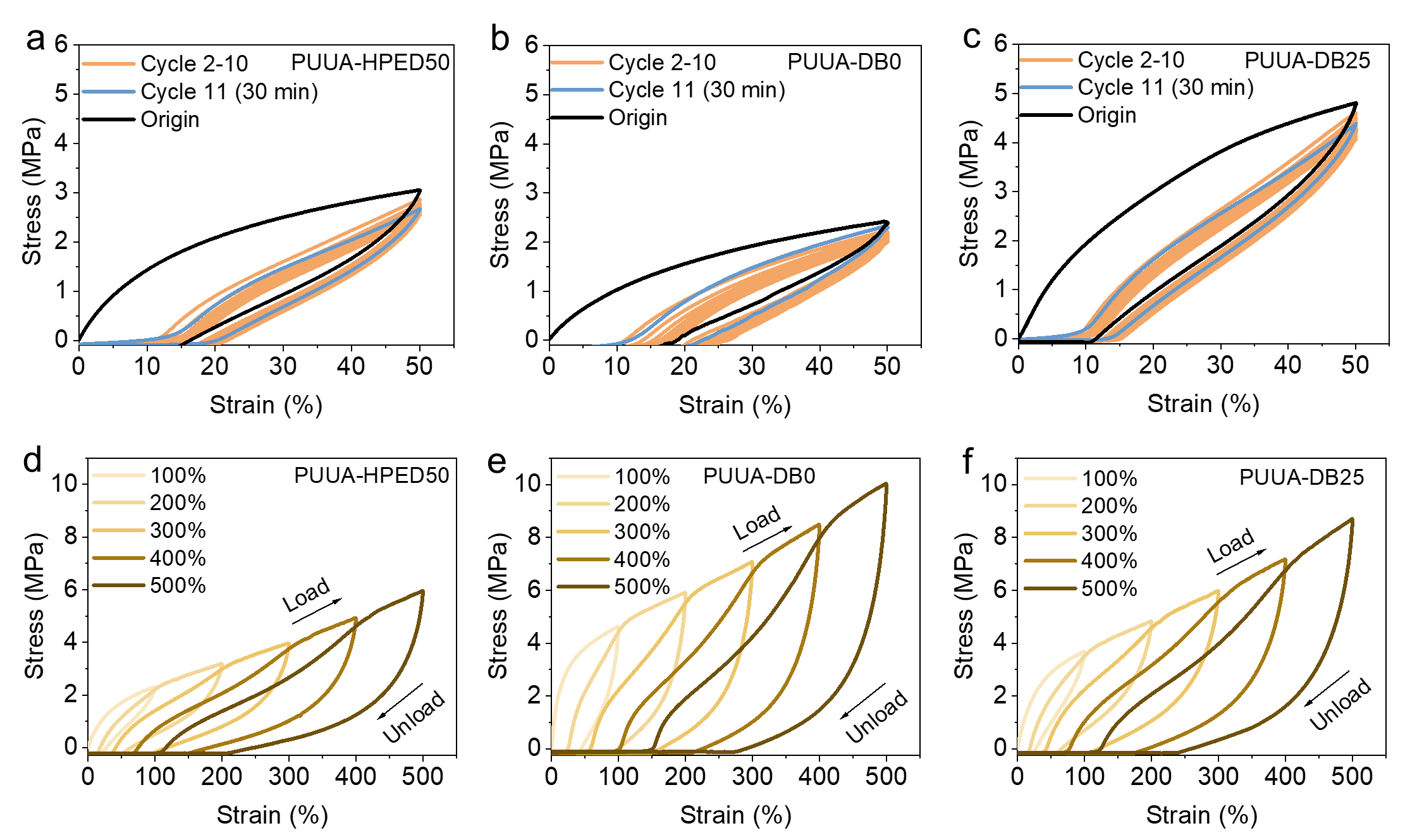


**Figure S8.** Stress-strain curves of the loading-unloading cycle for (**a**) PUUA-HPED50, (**b**) PUUA-DB0 and (**c**) PUUA-DB25. Stress-strain curves of the loading-unloading cycle at different large deformations for (**d**) PUUA-HPED50, (**e**) PUUA-DB0 and (**f**) PUUA-DB25.


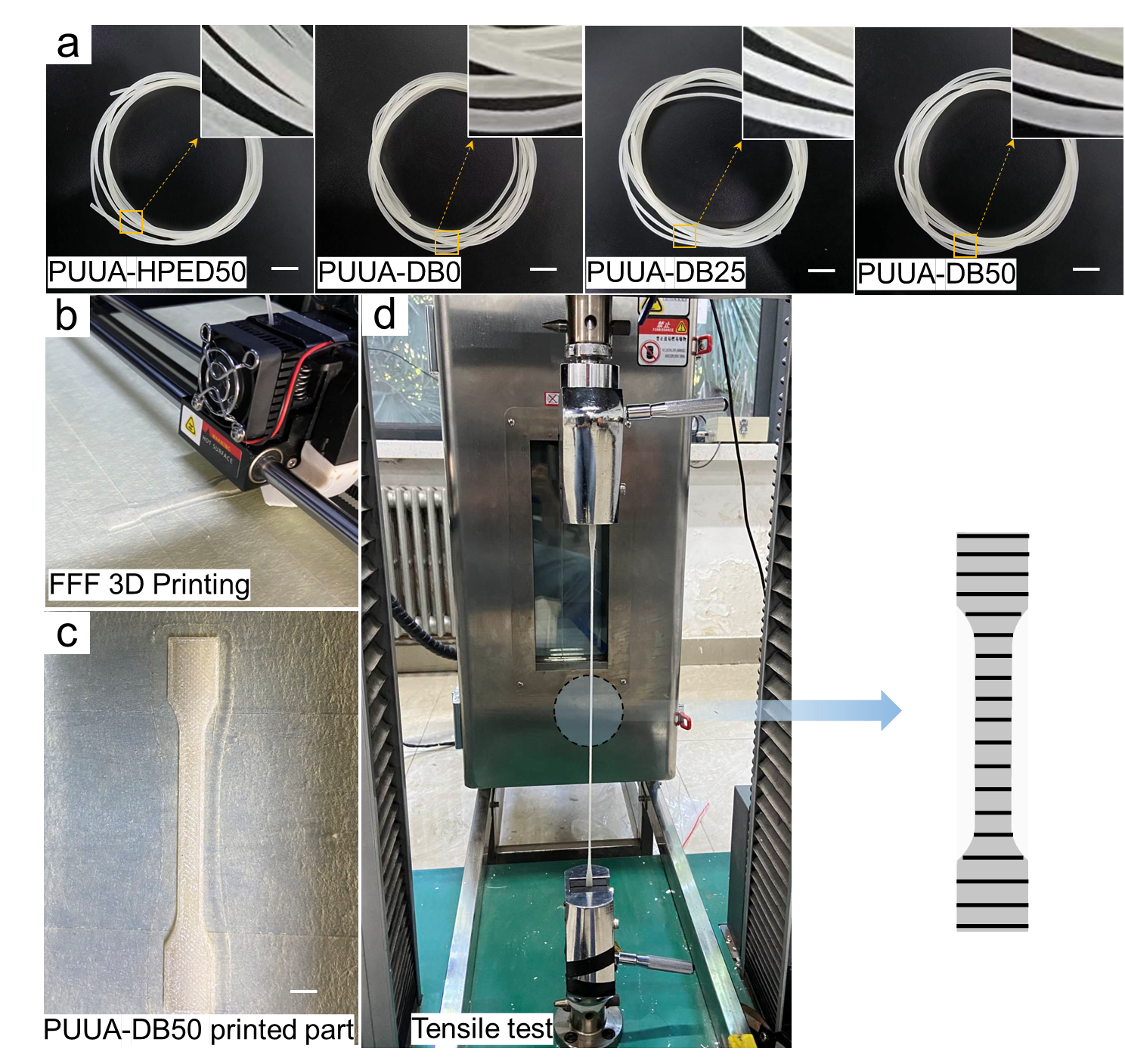


**Figure S9.** (**a**) Photograph images of PUUA-DBs filaments. (**b**) Fused filament fabrication process of PUUA-DBs. (**c**) Image of PUUA-DB printed part. (**d**) Tensile testing of sample in the 0° configuration (scale bars: 5 mm).


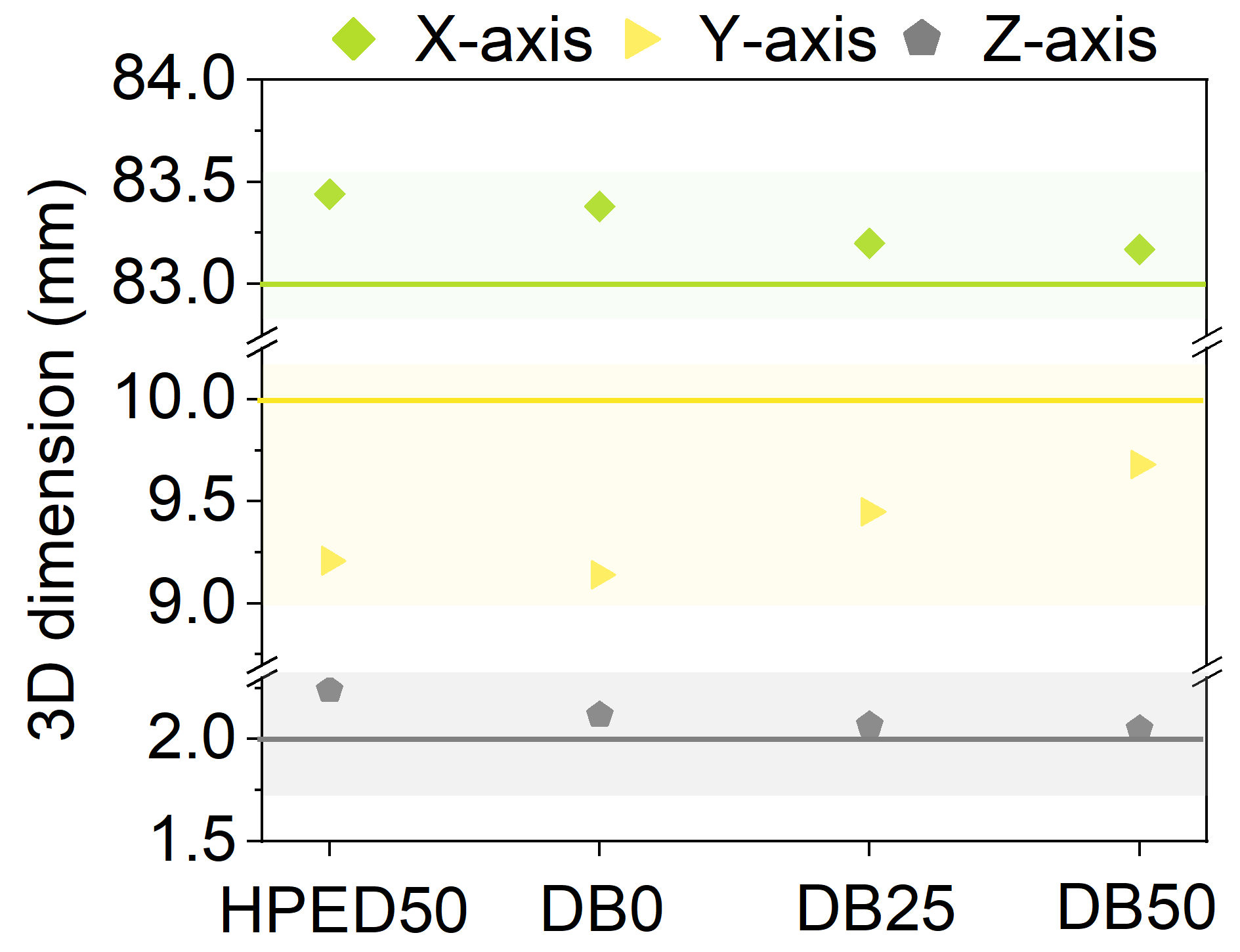


**Figure S10.** 3D dimensions (solid lines) and deviations (points) of FDM printed parts.


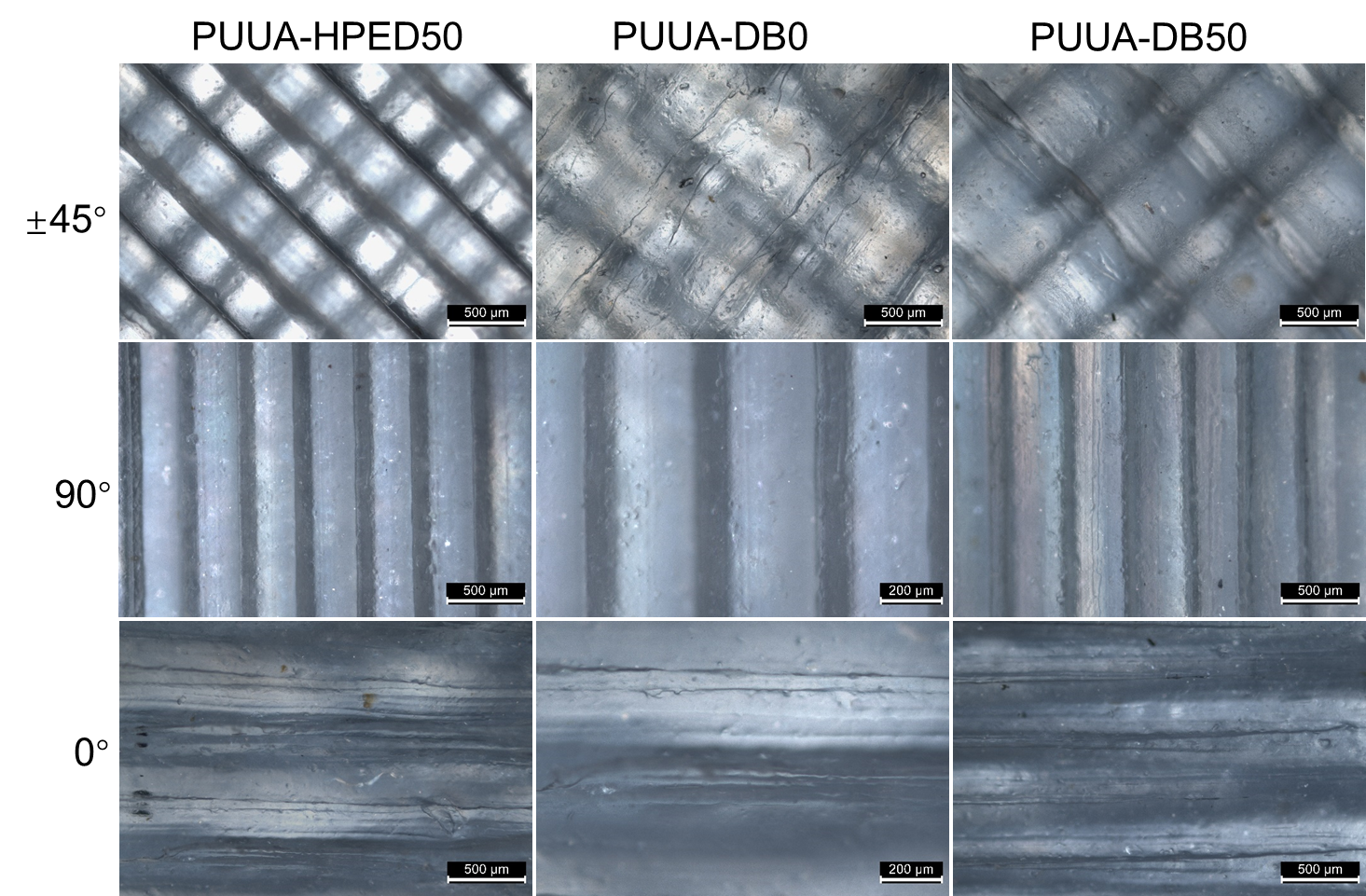


**Figure S11.** Images of PUUA-DB printed parts with 0°, ±45° and 90° raster angles. Scale bars: 500 μm.


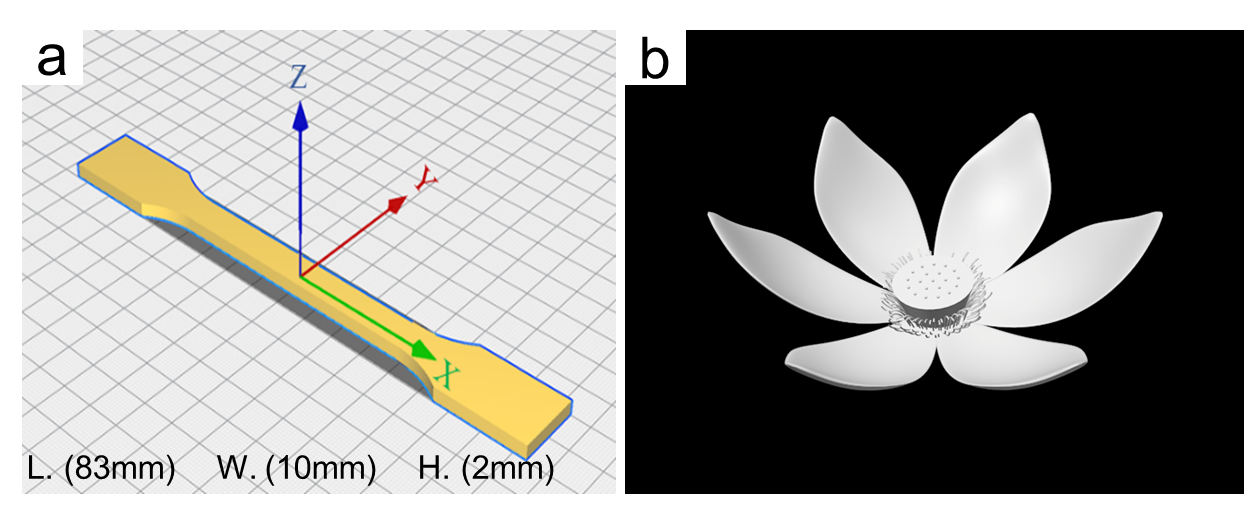


**Figure S12.** The digital files of dumbbell shaped part (**a**) and lotus flower (**b**) model.


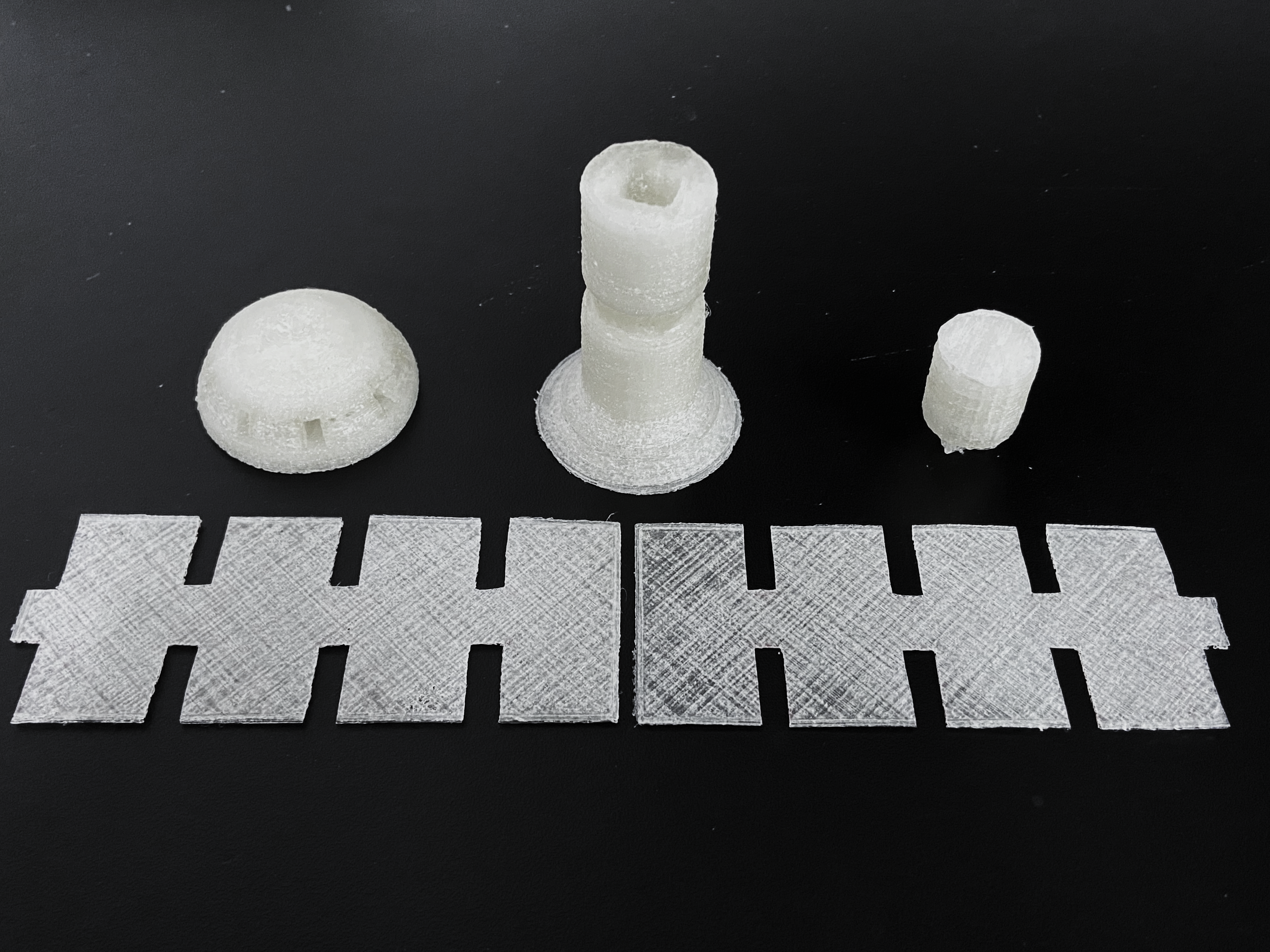


FDM-printed spacecraft parts

**Figure S13.** Images of FDM-printed spacecraft parts with PUUA-DB50 wire.


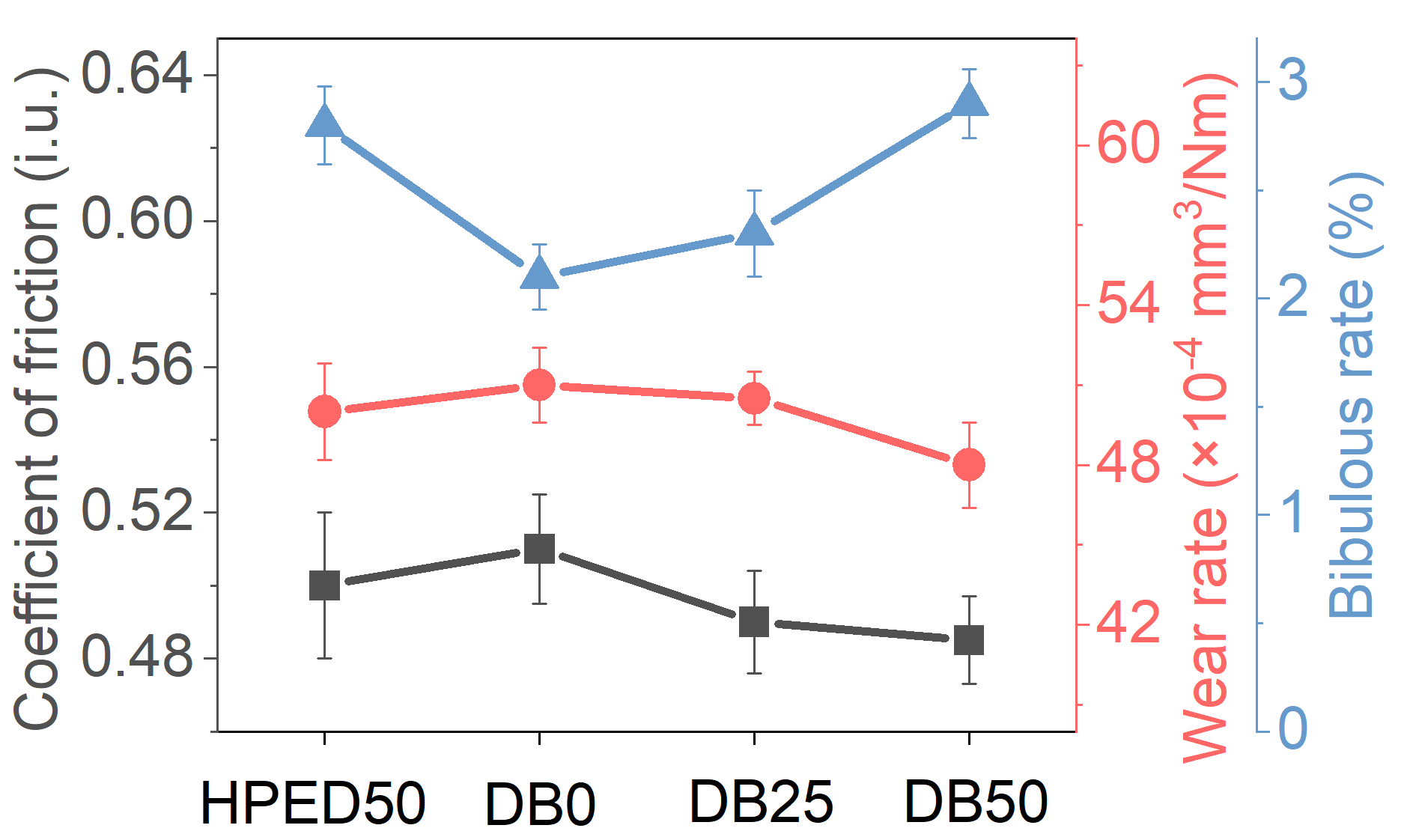


**Figure S14.** The coefficient of friction, wear rate and bibulous rate of PUUA-DB elastomers.

**Discussion:** This material exhibits inherently superior wear resistance and a low bibulous rate. Compared to PUUA-DB0, the friction coefficient (0.485) and wear rate (48.0×10^-4^ mm^3^ Nm^-1^) of PUUA-DB50 both slightly decreased (**Equation 11** and **Equation 12**). This improvement in wear resistance could be attributed to the higher cohesive energy (*E*_cohesive_) of PUUA-DB50 (**Figure 2e, f**), which mitigated surface stress concentration on the friction interface between the polymer and stainless steel counterfaces. Additionally, while PUUA-DB50 demonstrates stronger hydrogen bond interactions compared to PUUA-DB0, leading to a slight increase in water absorption, but the rate remains below 3 wt.%.


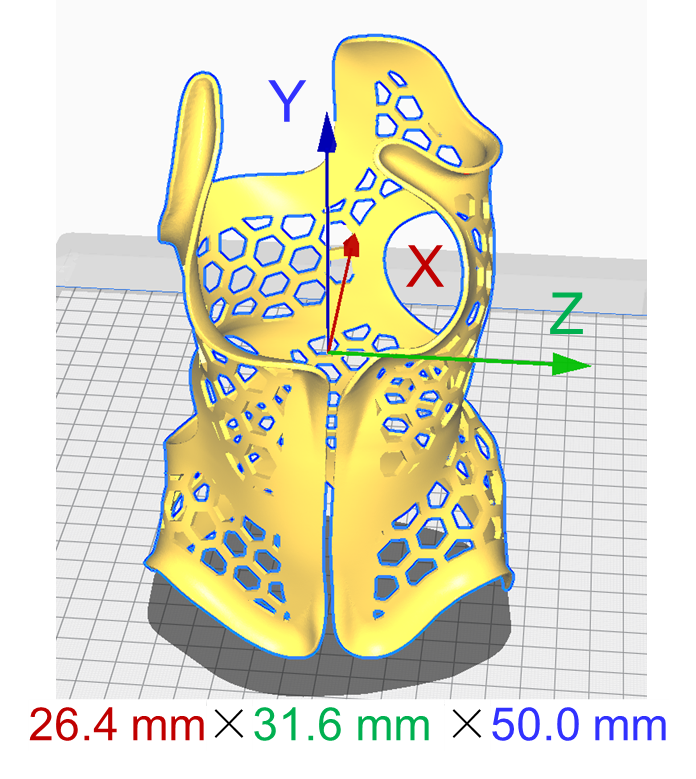


**Figure S15.** The digital file of spinal orthosis model.


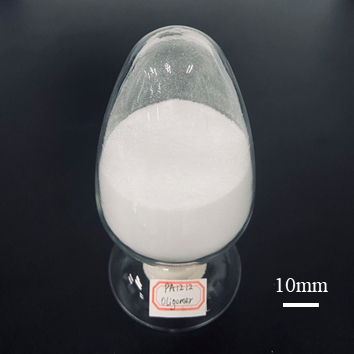


**Figure S16.** Polyamide 1212 oligomer powders.

**Supplementary Methods**

**Quantum chemistry calculation:**

All quantum-chemistry calculations were implemented by the Gaussian 09W suite of programs.^[20]^ In the calculation of interaction energy, the geometries conformation of PUUA-DB were optimized by using density functional theory (DFT) at B3LYP/Def2-TZVP^[21]^ level corrected with Grimme dispersion^[22]^ and BJ damping.^[23]^ In order to obtain accurate binding energy of hydrogen bond, the Counterpoise correction was simultaneously adapted to solve the problem of basis set superposition error (BSSE).^[24]^ The interaction energy (∆*E*_H_, kJ mol^−1^) was defined as follows:

$\Delta E_{H}=E_{AB}-E_{A}-E_{B}+E_{BSSE}$ (1)

where ∆*E*_H_, *E*_AB_, *E*_A_ and *E*_B_ are the interaction energy of hydrogen bond, hydrogen bond complex and monomers (A, B), *E*_BSSE_ is the basis-set superposition error.

**Molecular Dynamics Simulations:**

All-atom Molecular dynamics (MD) simulations were conducted by the Materials Studio (Accelrys, Inc.).

*Model Buildings:* First, a molecular chain with certain chemical structure were built and each of molecular chain contains two PA1212 segments, two PTMG and ED2003 segments, a PETMP segment and eight MDI segments. The number of repeating units in the PA1212, PTMG and ED2003 segments are 1, 14 and 20, respectively. Then, amorphous molecular model containing ten chains was built by Amorphous Cell Module and the model was subsequently minimized in order to eliminate the inappropriate geometry.

*Detailed MD simulations:* The simulations were conducted by the Forcite Module with COMPASSII force field, and the atomic point charges reproducing the electrostatic potential were implemented by using the Gasteiger method. In addition, electrostatic interaction and van der Waals force are based on Ewald and Atom methods, respectively. Firstly, the MD simulation was performed under NPT (P = 1 bar, T = 298 K) ensemble for 5 × 10^5^ steps with a time step of 0.5 fs, aiming to obtain an equilibrium configuration. The temperature was controlled by the Nosé-Hoover thermostat and the pressure were coupled by Berendsen barostat. The structures were annealed via one annealing cycle including a linear heating process from 298 K to 500 K and a linear cooling process from 500 K to 298 K.^[25]^

The cohesive energy per chain (*E*_cohesive_) was calculated by equation 2:

$E_{cohesive}=\frac{\sum_{i=1}^{10} E_{nb}^{isolated}\left(i\right)-E_{nb}^{10}}{10}$ (2)

where *E*isolated nb(*i*) is the average non-bonded energy of an isolated polymer chain in vacuum, and *E*10 nbis the average non-bonded energy of the simulation cell with ten polymer chains.^[26]^

For the PUUA-DB0 system, $E_{nb}^{10}$ = −25064.03 kJ mol^−1^ and $E_{nb}^{isolated}$ = -805.11 kJ mol^−1^.

For the PUUA-DB50 system, $E_{nb}^{10}$ = −30005.17 kJ mol^−1^ and $E_{nb}^{isolated}$ = -1132.45 kJ mol^−1^.

**Nuclear magnetic resonance spectroscopy (^1^H and** **^13^C NMR)**

The ^1^H and ^13^C NMR specta were obtained on Bruker Avance 300 (Germany), with trifluoroacetic acid-D used as the solvent.

## 3D Printing

The 3D printed objects were printed on a F5060 3D printer. The nozzle diameter was 0.4 mm. The 3D models were designed by SolidWorks, which was then imported into Ultimaker Cura 4.10.0 for parameter setting and G-code scripts generation. The printing temperature was 200 °C, and the build platform temperature was 60 °C. The printing speed was set as 40 mm s^−1^ and the layer height was 0.1 mm. All the samples were printed with 100% infill density (**Table S3**). For a dumbbell-shaped sample, the pattern consisted of alternating 0°, ±45° and 90° infill angles. The +45° and −45° patterns were printed alternately layer by layer (**Figure 1**c), while the 0° and 90° patterns were printed with corresponding infill angles.

**Determination of the stress relaxation**

Stress relaxation experiments were performed on dynamic mechanical analysis (TA-Q800 DMA) instrument with the controlled-force mode.^[27]^ First, a 30 mm × 6 mm × 0.5 mm rectangular sample was heated and stabilized at the set temperature, and then stretched to strain of 15%. Finally, the ratio of the instantaneous stress (*σ*) to the initial stress (*σ*_0_) was measured by DMA. The characteristic relaxation times *(τ**) were plotted as a function of the reciprocal of temperature and fitted using the Arrhenius relationship in equation 3:^[28]^

$\ln\tau^{*}\left(T\right)=\ln\tau_{0}+\frac{E_{a}}{RT}$ (3)

where *τ_0_* is the characteristic relaxation time at infinite *T*, that is the time for the modulus to relax to e^−1^ (37%) of the initial modulus. *E*_a_ represents the activation energy of exchange reaction, *R* is the universal gas constant (8.314 J mol^−1^ K^−1^) and *T* ranges from 303 K to 403 K during the stress relaxation experiment.

**Dual-shape memory**

FDM-printed PUUA-DB samples (30 mm × 6 mm × 0.5 mm) were heated to 50 °C after applying a preload of 0.01N and an initial deformation strain of 0.3%. After stabilized at 50 °C for 5 min, the sample was stretched to 50% at a strain rate of 25%/min, and then cooled to −20 °C at a rate of 5 °C min^−1^ and kept isothermally for 5 min. The temporary shape was fixed after the external force was removed and it was kept at −20 °C for another 5 min. Finally, the sample was heated to 50 °C at a rate of 5 °C min^−1^ and kept at this temperature for 10 min to let it recover to the initial shape. The shape-fixing ratio (*R*_f_) and shape-recovery ratio (*R*_r_) of the samples were defined as follows:

 (4)

 (5)

where *ε*_0_ represents initial strain after preloading in each cycle, *ε*_load_ is the maximum strain under loading, *ε*_unload_ is the fixed strain after cooling and removal of the external force, and *ε*_r_ is the residual strain after recovery.

**Reversible two-way shape memory**

FDM-printed PUUA-DB samples (30 mm × 6 mm × 0.5 mm) were first heated to 50 °C, and then stretched to a strain 100%. The sample was cooled to *T*_low_ = −20 °C, followed by the removal of the external force. The temperature was reversibly varied between *T*_high_ (30 °C) and *T*_low_ under the stress-free condition and the sample shifted automatically between the initial and stretched state. The heating and cooling rates were both 5 °C min^−1^. Here, three consecutive cycles were carried out to evaluate the repeatability of the reversible two-way shape memory effect, which was quantitatively characterized by calculating the reversible response strain (*ε*_2w_), actuation magnitude (*R*_act_) and recovery magnitude (*R*_r, 2w_) as equations 6, 7 and 8, respectively:

$\varepsilon_{2\text{w}}=\left(\varepsilon_{low}-\varepsilon_{0}\right)\times100\%$ (6)

$R_{\text{act}}\text{=}\frac{\varepsilon_{2\text{w}}}{\varepsilon_{\text{unload}}-\varepsilon_{\text{i}}}\times100\text{\%}$ (7)

$R_{r,2w}=\frac{\varepsilon_{low}-\varepsilon_{high}}{\varepsilon_{low}-\varepsilon_{0}}\times100\%$ (8)

Where *ε*_0_ is the initial strain at high temperature *T*_high_ in each cycle, *ε*_low_ is the maximum strain at low temperature *T*_low_ in each cycle, *ε*_high_ is the residual strain after reheating in each cycle, *ε*_i_ and *ε*_unload_ is the initial strain and the fixed strain after cooling and removal of the external force in a pre-programming step, respectively.

**Quadruple-shape memory**

Quadruple-shape memory effects were measured by DMA with tensile mode and the strain rate mode. First, the film sample with initial shape (A) was stretched to a strain of 25% at the temperature *T*_high_, and then the first temporary shape (B) was fixed after cooling to *T*_2, mid_ and removal of the external force. Secondly, the sample was further stretched to 50% at the temperature *T*_2, mid_ and the second temporary shape (C) was fixed at *T*_1, mid_ after the external force was removed. Finally, the sample was stretched to 75% again at *T*_1, mid_ and the third temporary shape (D) was fixed at *T*_low_ after unloading. When the sample was reheated to *T*_1, mid_, *T*_2, mid_ and *T*_high_ successively, the shapes C, B and A were obtained in order. In this quadruple shape memory procedure, the strain applied on the sample increased gradually and the temperature decreased gradually (*T*_high_ > *T*_2, mid_ > *T*_1, mid_ > *T*_low_) in each shape fixing stage. The *R*_f_ and *R*_r_ of each shape memory stage in the quadruple-shape memory process were calculated by equation 9 and equation 10, respectively:

$R_{\text{f(x}\to\text{y)}}\text{=}\frac{\varepsilon_{\text{y,unload}}-\varepsilon_{\text{x,unload}}}{\varepsilon_{\text{y,load}}-\varepsilon_{\text{x,unload}}}\times100\text{\%}$ (9)

$R_{\text{r(y}\to x\text{)}}\text{=}\frac{\varepsilon_{\text{y,unload}}-\varepsilon_{\text{x,r}}}{\varepsilon_{\text{y,unload}}-\varepsilon_{\text{x,unload}}}\times100\text{\%}$ (10)

where x and y denote two shapes in one shape memory stage of one cycle, *ε*_y, load_ is the maximum strain under loading, *ε*_x, unload_ and ε_y, unload_ are fixed strain after cooling to a lower temperature and unloading in last and this stage, respectively, and *ε*_x, r_ is the residual strain after recovery in this stage.

**Sliding friction and wear tests**

Following Chinese standard GB/T 33960-2016, the friction and wear performance were measured using a sliding friction and wear tester (M-200, China). The rotational speed of the metal steel ring (45^#^) was set to 200 rpm, with an applied load of 196 N and surface roughness R_a_ ≤ 0.4 μm. The friction coefficient (*μ*) and volumetric wear rate (*W*) were calculated using equation 11 and equation 12, respectively:

$\mu=\frac{M}{RF_{N}}$ (11)

$W=d\left[\frac{\pi R^{2}}{180}\arcsin\left(\frac{b}{2R}\right)-\frac{b}{2}\sqrt{R^{2}-\frac{b^{2}}{4}}\right]/F_{N}L$ (12)

where *M* denotes the friction torque (Nm). *R* is the radius of the metal ring (mm). *F_N_* denotes the normal load (N). *b* and *d* represent the width of the wear scar and sample, respectively (mm). *L* is the total sliding distance (m), and *W* is the volumetric wear rate, mm^3^ Nm^-1^.

**Water-uptake test**

First, the specimens (60 mm × 60 mm× 2 mm) were dried under vacuum at 80 °C to a constant weight. Then the specimens were immersed in a baker ﬁlled with deionized water at 23.0 ± 0.5 °C. Following a 48-hour soaking period, removed and the excess surface water was promptly blotted with tissue paper before reweighing. The bibulous rate was determined by tracking the increase in the weight.

References

[1] a)P. Fu, H. Li, J. Gong, Z. Fan, A. T. Smith, K. Shen, T. O. Khalfalla, H. Huang, X. Qian, J. R. McCutcheon, L. Sun, *Prog. Polym. Sci.* **2022**, 126, 101506; b)J. J. Koh, X. Q. Koh, J. Y. Chee, S. Chakraborty, S. Y. Tee, D. Zhang, S. C. Lai, J. C. C. Yeo, J. W. J. Soh, P. Li, S. C. Tan, W. Thitsartarn, C. He, *Adv. Sci.* **2024**, 11, 2402390.

[2] E. Yarali, M. J. Mirzaali, A. Ghalayaniesfahani, A. Accardo, P. J. Diaz-Payno, A. A. Zadpoor, *Adv. Mater.* **2024**, 36, 2402301.

[3] B. Liu, H. Li, F. Meng, Z. Xu, L. Hao, Y. Yao, H. Zhu, C. Wang, J. Wu, S. Bian, W. W. Lu, W. Liu, H. Pan, X. Zhao, *Nat. Commun.* **2024**, 15, 1587.

[4] S. Shaffer, K. Yang, J. Vargas, M. A. Di Prima, W. Voit, *Polymer* **2014**, 55, 5969.

[5] W. Yang, W. Zhao, Q. Li, H. Li, Y. Wang, Y. Li, G. Wang, *ACS Appl. Mater. Interfaces* **2020**, 12, 3928.

[6] J. E. Lee, S. J. Park, Y. Son, K. Park, S. Park, *Addit. Manuf.* **2021**, 43, 101995.

[7] A. Miller, G. Warner, G. Owolabi, *J. Mater. Eng. Perform.* **2021**, 30, 6673.

[8] N. P. Levenhagen, M. D. Dadmun, *ACS Appl. Polym. Mater.* **2019**, 1, 876.

[9] Q. Zhao, W. Zou, Y. Luo, T. Xie, *Sci. Adv.*, 2, 1501297.

[10] C. Cui, L. An, Z. Zhang, M. Ji, K. Chen, Y. Yang, Q. Su, F. Wang, Y. Cheng, Y. Zhang, *Adv. Funct. Mater.* **2022**, 32, 2203720.

[11] Y. Lai, X. Kuang, P. Zhu, M. Huang, X. Dong, D. Wang, *Adv. Mater.* **2018**, 30, 1802556.

[12] K. Yang, J. C. Grant, P. Lamey, A. Joshi-Imre, B. R. Lund, R. A. Smaldone, W. Voit, *Adv. Funct. Mater.* **2017**, 27, 1700318.

[13] Q. Jin, R. Du, H. Tang, Y. Zhao, W. Peng, Y. Li, J. Zhang, T. Zhu, X. Huang, D. Kong, *Angew. Chem.* **2023**, 135, 202305282.

[14] F. Sun, L. Liu, T. Liu, X. Wang, Q. Qi, Z. Hang, K. Chen, J. Xu, J. Fu, *Nat. Commun.* **2023**, 14, 130.

[15] a)J. Wang, S. Hu, B. Yang, G. Jin, X. Zhou, X. Lin, R. Wang, Y. Lu, L. Zhang, *ACS Appl. Mater. Interfaces* **2022**, 14, 1994; b)Z. Fang, H. Mu, Z. Sun, K. Zhang, A. Zhang, J. Chen, N. Zheng, Q. Zhao, X. Yang, F. Liu, J. Wu, T. Xie, *Nature* **2024**, 631, 783.

[16] a)Tzoganakis, Costas, *Adv. Polym. Technol.* **1989**, 9, 321; b)Y. Qu, Y. Chen, X. Ling, J. Wu, J. Hong, H. Wang, Y. Li, *Macromolecules* **2022**, 6, 2824.

[17] Z. Li, S. Mei, L. Luo, S. Li, X. Chen, Y. Zhang, W. Zhao, X. Zhang, G. Shi, Y. He, Z. Cui, P. Fu, X. Pang, M. Liu, *Macromol. Rapid Commun.* **2022**, 44, 2200693.

[18] Z. Li, Z. Cao, Q. Zhao, S. Mei, Y. Zhang, W. Zhao, X. Li, X. Zhang, Z. Cui, P. Fu, X. Pang, M. Liu, *Chem. Eng. J.* **2024**, 485, 149933.

[19] K. Nojima, K. Sanui, N. Ogata, N. Yui, K. Kataoka, Y. Sakurai, *Polymer* **1987**, 28, 1017.

[20] M. J. Frisch, G. W. Trucks, H. B. Schlegel, G. E. Scuseria, M. A. Robb, J. R. Cheeseman, G. Scalmani, V. Barone, G. A. Petersson, H. Nakatsuji, X. Li, M. Caricato, A. V. Marenich, J. Bloino, B. G. Janesko, R. Gomperts, B. Mennucci, H. P. Hratchian, J. V. Ortiz, A. F. Izmaylov, J. L. Sonnenberg, Williams, F. Ding, F. Lipparini, F. Egidi, J. Goings, B. Peng, A. Petrone, T. Henderson, D. Ranasinghe, V. G. Zakrzewski, J. Gao, N. Rega, G. Zheng, W. Liang, M. Hada, M. Ehara, K. Toyota, R. Fukuda, J. Hasegawa, M. Ishida, T. Nakajima, Y. Honda, O. Kitao, H. Nakai, T. Vreven, K. Throssell, J. A. Montgomery Jr., J. E. Peralta, F. Ogliaro, M. J. Bearpark, J. J. Heyd, E. N. Brothers, K. N. Kudin, V. N. Staroverov, T. A. Keith, R. Kobayashi, J. Normand, K. Raghavachari, A. P. Rendell, J. C. Burant, S. S. Iyengar, J. Tomasi, M. Cossi, J. M. Millam, M. Klene, C. Adamo, R. Cammi, J. W. Ochterski, R. L. Martin, K. Morokuma, O. Farkas, J. B. Foresman, D. J. Fox, Wallingford, CT 2016.

[21] F. Weigend, R. Ahlrichs, *PCCP* **2005**, 7, 3297.

[22] S. Grimme, J. Antony, S. Ehrlich, H. Krieg, *J. Chem. Phys.* **2010**, 132, 154104.

[23] S. Grimme, S. Ehrlich, L. Goerigk, *J. Comput. Chem.* **2011**, 32, 1456.

[24] S. F. Boys, F. Bernardi, *Mol. Phys.* **1970**, 19, 553.

[25] a)T. Zheng, T. Li, J. Shi, T. Wu, Z. Zhuang, J. Xu, B. Guo, *Macromolecules* **2022**, 55, 3020; b)Z. Li, Y. Zhu, W. Niu, X. Yang, Z. Jiang, Z. Lu, X. Liu, J. Sun, *Adv. Mater.* **2021**, 33, 2101498.

[26] Z. Li, Z. Lu, Z. Sun, Z. Li, L. An, *J. Phys. Chem. B* **2007**, 111, 5934.

[27] C. Cui, X. Chen, L. Ma, Q. Zhong, Z. Li, A. Mariappan, Q. Zhang, Y. Cheng, G. He, X. Chen, Z. Dong, L. An, Y. Zhang, *ACS Appl. Mater. Interfaces* **2020**, 12, 47975.

[28] M. M. Obadia, B. P. Mudraboyina, A. Serghei, D. Montarnal, E. Drockenmuller, *J. Am. Chem. Soc.* **2015**, 137, 6078.

**Description of Additional Supplementary Files**

**File Name:** **Supplementary Movie 1**

**Description:** Fused filament fabrication process of PUUA-DB50.

**File Name: Supplementary Movie 2**

**Description:** The dual shape memory effect of PUUA-DB50 printed part.

**File Name: Supplementary Movie 3**

**Description:** The reversible two-way shape memory effect of PUUA-DB50 printed part.

**File Name: Supplementary Movie 4**

**Description:** Fused deposition modeling of spinal orthosis with PUUA-DB50.

**File Name: Supplementary Movie 5**

**Description:** The dual shape memory effect of 4D-printed spinal orthosis with PUUA-DB50.
